# The Arabidopsis *WRR4A* and *WRR4B* paralogous NLR proteins both confer recognition of multiple *Albugo candida* effectors

**DOI:** 10.1111/nph.18378

**Published:** 2022-08-07

**Authors:** Amey Redkar, Volkan Cevik, Kate Bailey, He Zhao, Dae Sung Kim, Zhou Zou, Oliver J. Furzer, Sebastian Fairhead, M. Hossein Borhan, Eric B. Holub, Jonathan D. G. Jones

**Affiliations:** ^1^ The Sainsbury Laboratory University of East Anglia Norwich NR4 7UH UK; ^2^ Department of Botany Savitribai Phule Pune University Ganeshkhind Pune 411007 India; ^3^ The Milner Centre for Evolution, Department of Biology and Biochemistry University of Bath Bath BA2 7AY UK; ^4^ Department of Biology University of North Carolina Chapel Hill NC 27599 USA; ^5^ School of Life Sciences Warwick Crop Centre, University of Warwick Wellesbourne CV35 9EF UK; ^6^ Agriculture and Agri‐Food Canada 107 Science Place Saskatoon SK S7N 0X2 Canada; ^7^ Present address: State Key Laboratory of Biocatalysis and Enzyme Engineering Hubei University Wuhan 430062 China

**Keywords:** *Albugo candida*, Arabidopsis, effectors, hypersensitive response, oomycete, recognition, TIR–NLR, *WRR4*

## Abstract

- The oomycete *Albugo candida* causes white blister rust, an important disease of Brassica crops. Distinct races of *A. candida* are defined by their capacity to infect different host plant species. Each *A. candida* race encodes secreted proteins with a CX_2_CX_5_G (‘CCG’) motif that are polymorphic and show presence/absence variation, and are therefore candidate effectors*.*
- The *White Rust Resistance 4* (*WRR4*) locus in *Arabidopsis thaliana* accession Col‐0 contains three genes that encode intracellular nucleotide‐binding domain leucine‐rich repeat immune receptors. The Col‐0 alleles of *WRR4A* and *WRR4B* confer resistance to multiple *A. candida* races, although both *WRR4A* and *WRR4B* can be overcome by the Col‐0‐virulent race 4 isolate AcEx1.
- Comparison of CCG candidate effectors in avirulent and virulent races, and transient co‐expression of CCG effectors from four *A. candida* races in *Nicotiana* sp. or *A. thaliana*, revealed CCG effectors that trigger WRR4A‐ or WRR4B‐dependent hypersensitive responses.
- We found eight WRR4A‐recognised CCGs and four WRR4B‐recognised CCGs, the first recognised proteins from *A. candida* for which the cognate immune receptors in *A. thaliana* are known. This multiple recognition capacity potentially explains the broad‐spectrum resistance to several *A. candida* races conferred by *WRR4* paralogues. We further show that of five tested CCGs, three confer enhanced disease susceptibility when expressed *in planta*, consistent with *A. candida* CCG proteins being effectors.

## Introduction

Plants activate powerful and effective defence responses upon pathogen perception by their cell‐surface or intracellular immune receptors (Jones & Dangl, 2006). Microbes can be recognised via cell‐surface pattern recognition receptors (PRRs) that detect pathogen‐associated molecular patterns (PAMPs) (Chinchilla *et al*., 2007; Heese *et al*., 2007) and activate pattern‐triggered immunity (PTI) (Boller & Felix, 2009). Pathogens in turn can suppress host recognition and defence via effectors (Toruño *et al*., 2016), which function either in the apoplast or are translocated into the host cell. These effectors may interfere with PAMP perception (Wawra *et al*., 2016) or PTI signalling (Fabro *et al*., 2011; Irieda *et al*., 2019). In turn, some pathogen effectors are detected by intracellular nucleotide‐binding domain leucine‐rich repeat (NLR) immune receptors (Jones & Dangl, 2006), leading to the activation of effector‐triggered immunity (ETI), which often culminates in a hypersensitive cell death response (HR) that restricts pathogen invasion (Jones & Dangl, 2006). Nucleotide‐binding domain leucine‐rich repeats carry either a Toll/Interleukin 1/Resistance (TIR) domain (TIR–NLRs, or TNLs) or a coiled‐coil (CC) domain at their N‐termini (Jones & Dangl, 2006).

Effector detection by NLRs activates defences and thwarts pathogen growth (Armstrong *et al*., 2005; Rehmany *et al*., 2005). Effectors can be recognised by direct binding to an NLR, or indirectly by their interactions with a host protein, which is either ‘guarded’ by NLRs or has evolved to mimic a host target (a ‘decoy’) (Cui *et al*., 2015). Alternatively, some NLRs detect multiple sequence‐unrelated effectors through an integrated domain or by forming protein complexes (Sarris *et al*., 2015; Guo *et al*., 2018; Martel *et al*., 2020). Effector recognition by some NLRs can involve a post‐LRR (PL) domain that comprises a C‐terminal jelly‐roll and Ig‐like domain (C‐JID) that contributes to effector binding to form a resistosome, (a wheel‐like pentameric structure formed by oligomerisation) that upon activation activates defence (Ma *et al*., 2020; Martin *et al*., 2020). Understanding the molecular basis of such interactions by identifying recognised effectors in the pathogen is important for elevating crop disease resistance.

White blister rust in crop and wild Brassicaceae species is caused by oomycete pathogens in the genus *Albugo* (Holub *et al*., 1995; Voglmayr & Riethmüller, 2006; Choi *et al*., 2007). Dispersal is by dehydrated sporangiospores (Heller & Thines, 2009). Approximately 24 physiological races of *Albugo candida* have been defined, primarily based on which Brassicaceae species they infect (Hiura, 1930; Pound & Williams, 1963; Saharan & Verma, 1992). For example, *A. candida* races 2 (Ac2V), 7 (Ac7V) and 9 (AcBoT) infect *Brassica juncea*, *B. rapa* and *B. oleracea* (Gupta *et al*., 2018). Race 4 infects *Capsella bursa‐pastoris* and *Arabidopsis thaliana* (Fairhead, 2016) and can also infect *Camelina sativa* (Castel *et al*., 2021). Independent isolates of race 4 (AcEm2 and AcNc2) were found on *C. bursa‐pastoris* in Kent (UK; Borhan *et al*., 2008) and infected field‐grown *A. thaliana* plants in Norwich (UK; McMullan *et al*., 2015). Race 4 variant AcEx1 was isolated from wild *A. halleri* (Fairhead, 2016), which grows and sporulates on most *A. thaliana* accessions and on *C. sativa* (Castel *et al*., 2021). Jouet *et al*. (2019) verified that different physiological races of *A. candida* exhibit distinct host specificities within Brassicaceae. Genome comparisons by McMullan *et al*. (2015) revealed rare recombination events between these races.

*Albugo* sp. have a marked capacity to impose host immunosuppression (Cooper *et al*., 2008; Belhaj *et al*., 2017; Prince *et al*., 2017). *Albugo* infection enhances host susceptibility and enables growth of nonadapted fungal and oomycete pathogens (Cooper *et al*., 2008), including the potato late blight pathogen, *Phytophthora infestans* (Belhaj *et al*., 2017; Prince *et al*., 2017). The races studied in the work reported here are summarised in Table 1.

**Table 1:** Resistance and susceptibility phenotypes on adult leaves of Arabidopsis accessions to different *Albugo candida* races.

*Albugo candida* isolate	Race	Host	Infection phenotype on *A. thaliana*			
			Col‐0	Ws‐2	Col‐0‐*wrr4a‐6*	Col‐0‐*wrr4b*
Ac2V	Race 2	*Brassica juncea*	Resistant (GR)	Resistant (GR)	Resistant (GR)	Resistant (GR)
Ac7V	Race 7	*Brassica rapa*	Resistant (GR)	Resistant (GR)	Resistant (GR)	Resistant (GR)
AcBoT	Race 9	*Brassica oleracea*	Resistant (GR)	Resistant (GR)	Resistant (GR)	Resistant (GR)
AcNc2	Race 4	*Arabidopsis thaliana*	Resistant (GR)	Susceptible (S)	Resistant (NCR)	Resistant (GR)
AcEm2	Race 4	*Capsella bursa‐pastoris*	Resistant (GR)	Susceptible (S)	Resistant (NCR)	Resistant (GR)
AcEx1	Race 4	*Arabidopsis halleri*	Susceptible (S)	Susceptible (S)	Susceptible (S)	Susceptible (S)

Host specificity of different *A. candida* races and their observed infection phenotypes on different accession of *A. thaliana* (GR, green resistance; NCR, necrotic–chlorotic resistance; S, susceptibility). Host indicates the plant host from which the *A. candida* isolate was originally isolated.

Genome analysis of *Albugo laibachii* and *A. candida* revealed a class of secreted proteins that carry a ‘CHxC’ motif (Kemen *et al*., 2011; Furzer *et al*., 2022). Re‐sequencing of the Ac2V isolate of *A. candida* from *B. juncea* using long reads, revealed a *c*. two‐fold expansion of CHxC effector‐like proteins in *A. candida* compared with *A. laibachii* (Kemen *et al*., 2011). These are now reclassified as CX_2_CX_5_G and abbreviated to CCG effectors (Furzer *et al*., 2022). Every *A. candida* race has *c*. 80–100 CCG proteins that comprise *c*. 10% of the secretome (Furzer *et al*., 2022). *CCG* genes show signatures of diversifying selection and display elevated rates of pseudogenisation and presence/absence variation, consistent with their selection for diversity while maintaining virulence functions (Furzer *et al*., 2022). We address here the question of whether secreted *A. candida* CCG proteins are effectors, by evaluating whether any are recognised by *White Rust Resistance* (*WRR*) genes, and testing if some confer enhanced disease susceptibility.

Infection phenotypes conferred by *White Rust Resistance 4* (*WRR4*) are classified into resistant (green resistant, GR), partially resistant with chlorosis or necrosis but no pustules (necrotic–chlorotic resistant, NCR), and susceptible, with pustules (Susceptible, S; Cevik *et al*., 2019). *WRR4* from *A. thaliana* confers resistance to *A. candida* (Borhan *et al*., 2008). The Col‐0 locus contains three paralogues that encode TIR–NLR (TNL) immune receptors. The Col‐0 allele of *WRR4A* confers resistance to four *A. candida* races (Borhan *et al*., 2008), and resistance to race Ac2V in transgenic *B. juncea* (Borhan *et al*., 2010). The Col‐0 allele of *WRR4B* also confers resistance to *A. candida* races Ac2V, Ac7V and AcBoT (Cevik *et al*., 2019). Although resistance in Col‐0 functions against multiple *Brassica*‐infecting *A. candida* races, some variants of race 4 (e.g. AcEx1) can grow and sporulate on Col‐0, but with a chlorotic phenotype (Fairhead, 2016; Jouet *et al*., 2019). Some accessions, for example Oystese (Oy‐0), resist race 4 isolate AcEx1, due to an allele of *WRR4A* with a C‐terminal extension of 80 amino acids (Fairhead, 2016; Castel *et al*., 2021).

Leaves of *A. thaliana* accession Wassilewskija (Ws‐2) are resistant to *A. candida* race 2 and race 7*. WRR4A* in Ws‐2 carries deletions compared with Col‐0. Ws‐2 contains two divergent *WRR4* paralogues (Van de Weyer *et al*., 2019) and one of these (a Ws‐2 allele of *WRR4B*) confers resistance to *A. candida* race 2 (from *B. juncea*). Both Col‐0 and Ws‐2 alleles of *WRR4B* confer resistance in transgenic Brassicas to *A. candida* race 2 (Cevik *et al*., 2019). Allelic variation of TIR–NLR paralogues at the *WRR4* locus therefore provides multiple genes that could control white rust in major Brassica crops. Identifying *A. candida* effectors recognised by *WRR4A* and *WRR4B* would help choose the most effective transgene combinations for *Albugo* control.

Here, we compared the genomes of different races of *A. candida* with the goal of identifying the cognate recognised effectors for the Col‐0 alleles of *WRR4A* and *WRR4B*. We screened multiple CCG secreted proteins, mainly from *A. candida* races 2 and 4, using *Agrobacterium*‐mediated transient co‐expression to identify pairwise combinations of effector and NLR variant that activate an HR. Twelve CCG candidates – eight recognised by *WRR4A* and four by *WRR4B* – show activation of HR when transiently co‐expressed, and were validated for their recognition by a bombardment assay in Col‐0 wild‐type and mutants (*wrr4a‐6* or *wrr4b*) of *A. thaliana*. Several of these CCGs are absent and some show expression or allelic polymorphism in the Col‐0 virulent isolate AcEx1. For *WRR4A*‐recognised CCGs, the N‐terminal 100 amino acids are sufficient for recognition. Our data reveal that two distinct *WRR4* paralogues confer broad‐spectrum resistance by recognition of multiple CCG effectors across distinct clades in the *A. candida* CCG effectorome. Moreover, some of the CCGs confer enhanced susceptibility to another oomycete pathogen *Hyaloperonospora arabidopsidis* (*Hpa*), consistent with the idea that CCG secreted proteins are authentic *A. candida* effectors.

## Materials and Methods

### Plant material and growth conditions

Wild‐type and mutant *A. thaliana* accessions used in this study included Col‐0, Wassilewskija‐2 (Ws‐2), Col‐0_*wrr4a‐6* (Borhan *et al*., 2008), Col‐0_*wrr4b* (Cevik *et al*., 2019) and the recombinant inbred line (RIL) CW20 that was derived from a cross between Col‐5 × Ws‐2. In this RIL, *WRR4* locus is the only known *WRR* locus introgressed from Col‐5 (Fairhead, 2016). Seeds were sown directly on compost and were grown at 21°C, with 10 h : 14 h, light : dark regime, at 75% humidity. For *Nicotiana tabacum* and *N. benthamiana*, plants were grown on compost at 21°C, with cycles of 16 h : 8 h, light : dark, at 55% humidity.

### *Albugo candida* infection assay

The *A. candida* races described in this study were collected from field infections (McMullan *et al*., 2015) and maintained for subsequent pathogenicity assays. For leaf inoculations, previous leaf infections were suspended in water (*c*. 10^5^ spores ml^−1^) and incubated on ice for 30 min for releasing zoospores from sporangia. The spore suspension was then sprayed on plants using a Humbrol spray (Hornby Hobbies Ltd, Sandwich, UK) at *c*. 700 μl per plant. Plants were incubated at 4°C in the dark overnight for zoospore germination. Infected plants were kept under 10 h : 14 h, light : dark, 21°C : 16°C cycles. Phenotypes were monitored between 7–10 d post inoculation (dpi).

Sequential infection assay was carried out as described previously (McMullan *et al*., 2015). We developed *A. candida* race‐specific PCR primers unique to the tested *A. candida* isolates AcNc2 and AcEx1 (Supporting Information Table S1). Primers were designed to amplify these regions from genomic DNA extracted from each isolate. Primary inoculum was sprayed onto control and test plants. For AcEx1 *WRR4A*‐mediated defence suppression assays, both *A. thaliana* Ws‐2 and CW‐20 were inoculated, with water‐treated plants as mock control. At 7 dpi, a secondary infection with the avirulent *A. candida* isolate AcNc2 was performed on 50% of the plants while the remaining 50% were mock inoculated with water. The co‐inoculated plants were incubated for a further 8 dpi. Tissue was harvested and washed in sterile water to remove surface adhering spores, and flash frozen in liquid N_2_. DNA was prepared using a DNeasy plant mini kit (Qiagen).

### Selection of candidate CCG effector‐like proteins from *A. candida* for recognition assays

CCG proteins to be screened for recognition by *WRR4A* and *WRR4B* were selected by allelic comparison and based on presence/absence polymorphism or truncation in virulent isolates (Jouet *et al*., 2019; Furzer *et al*., 2022). We selected 30 candidate CCGs to screen with *WRR4A*, prioritising those either absent or truncated in *WRR4A*‐overcoming race 4 isolate AcEx1 and present across other *A. candida* races. To screen with *WRR4B*, we selected 13 candidate CCGs present in *B. juncea*‐infecting race Ac2V or present in Ac2V, Ac7V and AcBoT (*A. candida* races infecting Brassica crops) but absent or truncated in other Arabidopsis‐infecting *A. candida* races. All cloned candidate CCGs from different races and the outcome of our screening are shown in Table 2.

**Table 2:** CCG candidates cloned and tested in our analysis from the *Albugo candida* CCG effectorome.

CCG	Ac2V	Ac7V	AcBoT	AcNc2	AcEm2	AcEx1
CCG3						
CCG6						
CCG9						
CCG11						
CCG14						
CCG15						
CCG16						
CCG17						
CCG19						
CCG26						
CCG28						
CCG30						
CCG31						
CCG33						
CCG34						
CCG35						
CCG36						
CCG40						
CCG42						
CCG44						
CCG46						
CCG47						
CCG55						
CCG56						
CCG58						
CCG67						
CCG71						
CCG72						
CCG79						
CCG104						
CCG8						
CCG45						
CCG53						
CCG54						
CCG57						
CCG61						
CCG66						
CCG69						
CCG70						
CCG74						
CCG75						
CCG77						
CCG82						

	Cloned, tested and found to be recognised
	Cloned, tested and found to be delayed in recognition
	Cloned and tested, but not recognised
	Not tested
	Absent in the particular *A. candida* isolate

*CCG* effector genes from different *A. candida* races were cloned and tested in high‐throughput transient screens to identify the recognised effectors by broad‐spectrum NLR genes *WRR4A* and *WRR4B*.

To test the selected CCG effectors for *WRR4A* or *WRR4B* recognition, CCG effectors excluding the signal peptide were cloned from race 2 (Ac2V) or race 4 (AcEm2 or AcNc2) into an expression vector with 35S promoter and transformed into *Agrobacterium* for infiltration into *N. tabacum* or *N. benthamiana* leaves. Transient co‐delivery of CCG effector was performed either with green fluorescent protein (GFP) or red fluorescent protein (RFP) as negative control and with *WRR4A* or *WRR4B*. Effectors that trigger an HR when co‐expressed with the corresponding NLR were further validated by the luciferase eclipse assay by particle bombardment.

### Gene cloning and plasmid construction

Cloning of genes were carried out using the Uracil‐Specific Excision Reagent (USER) method (Geu‐Flores *et al*., 2007). Genes with 5′ and 3′ regulatory sequences were cloned into the LBJJ233‐OD vector prelinearised with *Pac*I and Nt.*Bbvc*I. For overexpression, plant genes and CCG effectors, were cloned into LBJJ234‐OD (containing a FAST‐Red selectable marker, CaMV 35S promoter and Ocs terminator) or pICH86966 or pICH86977 (containing a kanamycin selectable marker) (Table S1). Genes were C‐terminally tagged either with a His‐FLAG (HF tag) or a yellow fluorescent protein (YFP) tag. Briefly, the candidate CCG effector was PCR amplified from one of the *A. candida* races (AcNc2, AcEm2 or Ac2V) for the high‐throughput screen for *WRR4A‐*recognised CCGs and from race Ac2V for *WRR4B‐*recognised CCGs. Genomic DNA was used as a template with KAPA HiFi Uracil+ enzyme. To obtain mutant versions of CCG28^aa29–130^‐YFP carrying mutations in the CCG motif (CCG exchanged to AAG, CAA, CAG and AAA), site‐directed mutagenesis was performed with a Quikchange multisite‐directed mutagenesis kit (Stratagene, Santa Clara, CA, USA) following the manufacturer's instructions. A list of primers and vectors is indicated in Table S1. *WRR4A* and *WRR4B* were cloned in pICH86966. All plasmids were transformed into *Escherichia coli* electro‐competent cells, selected with appropriate antibiotics and purified using a Qiaprep spin miniprep kit (Qiagen). Positive clones were transformed in *Agrobacterium tumefaciens* strain GV3101 and used in infiltrations for transient expression experiments.

### Transient expression in *N. tabacum* or *N. benthamiana* leaves and cell death assay

*Agrobacterium tumefaciens* strains were streaked on selective medium and incubated at 28°C for 24 h. The streaked inoculum was transferred to liquid LB medium with the appropriate antibiotic and incubated at 28°C for 24 h in a shaking incubator at 200 rotations min^−1^ (rpm). The resulting cultures was centrifuged at 1000 ***g*** for 5 min and resuspended in infiltration buffer (10 mM MgCl_2_, 10 mM MES, 150 μM acetosyringone pH 5.6) at an OD_600_ of 0.4 (2 × 10^8^ cfu ml^−1^). For co‐expression, each bacterial suspension was adjusted to an OD_600_ of 0.4. The abaxial surfaces of 4‐wk‐old *N. tabacum* or 5‐wk‐old *N. benthamiana* were infiltrated with a 1‐ml needleless syringe. Cell death was phenotyped 2–4 d after infiltration. For the *WRR4B* recognition assay, macroscopic cell death phenotypes were scored according to the HR index modified from Segretin *et al*. (2014) based on an arbitrary scale ranging from 0 (no visible necrosis) to 6 (full necrosis).

### Particle bombardment in *A. thaliana* and luciferase assay

Transient protein expression in Arabidopsis leaves was performed by biolistic gene delivery. Here, 1.0‐μm tungsten particles (Bio‐Rad) were coated with the plasmids coding for the indicated *CCG* genes driven under the CaMV 35S promoter (Table S2) and/or empty luciferase plasmid. Bombardment was performed using a PDS‐1000/He system (Bio‐Rad) onto 4‐wk‐old Arabidopsis leaves from accession Col‐0 and Col‐0 *wrr4a‐6* or *wrr4b* mutants. After bombardment the leaves were incubated in vials with the leaf petiole immersed in water for 48 h post bombardment (hpb). The leaves were then frozen in liquid N_2_ and stored at −80°C until further processing.

For the luciferase assay a dual reporter luciferase assay system (Promega) was used and luciferase activity was measured following a protocol modified from Allen *et al*. (2004) previously used for GUS. Four transiently bombarded leaf events were pooled together and crushed in lysis buffer. The extract was centrifuged at 16 000 ***g*** for 10 min at 4°C. Next, 20 μl of the lysate was then dispersed in 96‐well plate and analysed on a Varioskan flash instrument by injecting 100 μl of luciferase assay reagent II, which includes substrate and reaction buffer. A 10 s read time was used to measure luciferase activity for each well. A higher luciferase activity was recorded for the luciferase‐bombarded leaves compared with the samples co‐delivered with the CCG candidate. The luciferase activity in the leaves co‐transformed with the CCG candidate effector dropped as a result of the cell death due to the candidate CCG recognition by the corresponding WRR4 but not in the Col‐0 *wrr4* mutants.

### Gene expression measurement by RT‐qPCR

For gene expression analysis, RNA was isolated from three biological replicates and used for reverse transcription quantitative PCR (RT‐qPCR) after cDNA synthesis. Briefly, RNA was extracted using the RNeasy plant mini kit (Qiagen) with the DNase treatment (Qiagen). Reverse transcription was carried out using SuperScript IV reverse transcriptase (ThermoFisher). Reverse transcription quantitative PCR was performed using CFX96 touch real‐time PCR (Bio‐Rad). Primers for qPCR analysis of different CCGs are listed in Table S1. Data were analysed using the double ΔΔ*C*_T_ method (Livak & Schmittgen, 2001) by calculating the relative expression of candidate CCG in relation to the *A. candida EF1α* as a housekeeping reference gene.

### Protein extraction and Western Blot

Protein was extracted from *Agrobacterium*‐infiltrated *N. benthamiana* leaves at 72 hpi as previously described (Sarris *et al*., 2015). Briefly, leaves were harvested, crushed in liquid N_2_, and extracted in GTEN buffer (10% glycerol, 100 mM Tris–HCl, pH 7.5, 1 mM EDTA, 150 mM NaCl, 5 mM 1,4‐dithiothreitol (DTT), 1× complete protease inhibitor mixture (Roche) and 0.2% (v/v) Nonidet P‐40). Next, 30 μl of the supernatant from the sample extract was used to elute the samples by boiling in loading buffer. Co‐immunoprecipitations were performed for 4 h at 4°C with gentle agitation, in the presence of 10 μl per 1 ml of protein extract of anti‐FLAG M2 affinity beads (A2220; Sigma‐Aldrich). Beads were washed four times in GTEN buffer. Samples were eluted by boiling in SDS‐PAGE loading buffer. For SDS‐PAGE, samples were heated for 10 min at 95°C for denaturation. After electrophoresis, separated proteins were transferred to PVDF (Merck, Darmstadt, Germany) membranes for immunoblotting. Membranes were blocked for 2 h in 5% nonfat milk, probed with horseradish peroxidase (HRP)‐conjugated antibodies for overnight. Chemiluminescence detection for proteins was carried out by incubating the membrane with developing reagents (Supersignal west‐pico or west‐femto), using an ImageQuant LAS 4000 instrument (GE Healthcare, Bio‐Sciences Corp., Piscataway, NJ, USA).

### Generation of transgenic Arabidopsis lines

To generate CCG‐expressing transgenic lines, candidate CCG effectors (both recognised and nonrecognised) were cloned under the 35S promoter without their secretion signal and with a C‐terminally tagged His‐FLAG (HF)‐tag in plasmid pICH86966 (containing a kanamycin selectable marker). Transgenic C‐terminally HF‐tagged CCG lines with CCG33, CCG44, CCG46, CCG47 and CCG71 were generated using the floral dip method (Clough & Bent, 1998) with *Agrobacterium tumefaciens* GV3101 carrying the candidate CCG construct. Transformation was carried out in Ws‐2 ecotype of *A. thaliana* that lacks functional *WRR4A*. *35S*_*Pro*_ *:GUS:HF* plants or nontransformed Ws‐2 plants were used as the negative control. Ws‐*eds1* was used as an enhanced susceptibility positive control.

### *Hpa* infection and quantification

Nontransformed, control and transgenic lines were tested for altered susceptibility to compatible *Hpa* Waco9 in cotyledons of 10‐d‐old Arabidopsis seedlings grown on soil. The plants were sprayed with a suspension of 1 × 10^5^ spores ml^−1^ and then placed in high (> 90%) humidity under a plastic dome. Sporulation on seedlings was scored at 7 dpi by quantifying the number of sporangiophores from 40 cotyledons per genotype using a Leica M165 FC fluorescence stereomicroscope connected to an EL6000 laser source. Only sporangiophores on the upper side of the cotyledons were counted.

### Statistical analysis

Statistical analysis was carried out using the GraphPad Prism 9.0 software (San Diego, CA, USA). The statistical test used is described in the figure legends.

## Results

### *WRR4A* ^Col^ ^‐0^ and *WRR4B* ^Col^ ^‐0/Ws‐2^ provide broad‐spectrum resistance to *A. candida* races

**Fig. 1:**
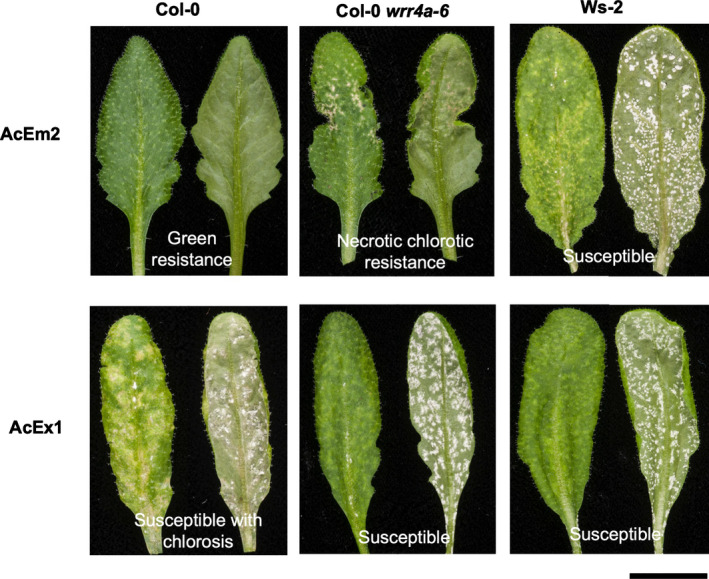
Resistant and susceptible phenotypes of *Arabidopsis thaliana* following inoculation of adult leaves with two different isolates of *Albugo candida* race 4. Arabidopsis Col‐0 is resistant to race 4 isolate AcEm2 (collected from *Capsella bursa‐pastoris*) and susceptible to race 4 isolate AcEx1 (collected from *A. halleri*) whereas Ws‐2 is susceptible to both isolates. Necrotic–chlorotic resistance (NCR) observed with the Col‐0‐*wrr4a‐6* mutant indicates that WRR4A is required for full green resistance (GR) to AcEm2. Five‐week‐old plants were spray inoculated with *A. candida* and incubated at 21°C; and then phenotyped 14 d after inoculation. Abaxial and adaxial photographs of the same leaf are shown. Bar, 1 cm.

To assess the resistance phenotypes of *WRR4A* to avirulent and virulent isolates of race 4 (AcEm2 and AcEx1), we tested wild‐type Col‐0 (*WRR4A* ^Col‐0^, *WRR4B* ^Col‐0^), Ws‐2 (*WRR4B* ^Ws2^) that lacks *WRR4A*, and a Col‐0 *wrr4a‐6* (*WRR4B* ^Col‐0^) T‐DNA mutant. *WRR4A* ^Col‐0^ confers green resistance to multiple *A. candida* races (Borhan *et al*., 2008; Cevik *et al*., 2019) including the race 2 isolate Ac2V and two highly similar race 4 isolates AcNc2 and AcEm2 (McMullan *et al*., 2015). However, Arabidopsis accession Ws‐2 that lacks *WRR4A* is fully susceptible to AcEm2, but the Col‐0‐*wrr4a‐6* mutant shows NCR rather than GR, due to the presence of additional *WRR* genes in Col‐0 (Fig. 1; Table 1). This suggests that *WRR4A* ^Col‐0^ enables GR to AcEm2 (Fig. 1). Moreover, *WRR4A* ^Col‐0^ confers weak resistance on Col‐0 that shows a chlorotic susceptibility to race 4 isolate AcEx1 (Prince *et al*., 2017; Fig. 1). By contrast, the Col‐0 *wrr4a‐6* mutant is fully susceptible to AcEx1 without any chlorosis, indicating that *WRR4A* ^Col‐0^ confers partial resistance to AcEx1, which is eventually overcome.

The *WRR4B* ^Col‐0/Ws‐2^ paralogues also confer resistance to Ac2V in *A. thaliana* and in transgenic *B. juncea* (Cevik *et al*., 2019). However, in Ws‐2, which lacks *WRR4A*, leaves exhibit full GR phenotype following inoculation with either Ac2V or Ac7V indicating that *WRR4B* ^Ws‐2^ confers full resistance to races 2 and 7 of *A. candida* (Table 1).

### Selecting candidate *A. candida* CCG effectors to test for recognition by *WRR4* paralogues

To identify CCG proteins that are recognised by *WRR4* paralogues, we analysed CCGs predicted from Illumina‐based genome assemblies of multiple *A. candida* races (AcNc2, AcEm2, AcEx1, Ac7V, AcBoT and Ac2V; McMullan *et al*., 2015; Jouet *et al*., 2019) as well as additional CCGs predicted from a long read assembly of Ac2V (Furzer *et al*., 2022). Each *A. candida* race carries *c*. 80–100 *CCG* genes that can vary by sequence and presence/absence variation (Jouet *et al*., 2019; Furzer *et al*., 2022). This enabled identification of candidates for functional screening of CCG effectors by transient expression, testing for *WRR* gene‐dependent HR. Such transient assays were used to identify recognised RxLR effectors from *Phytophthora infestans* (Vleeshouwers *et al*., 2008; Vleeshouwers *et al*., 2011).

**Fig. 2:**
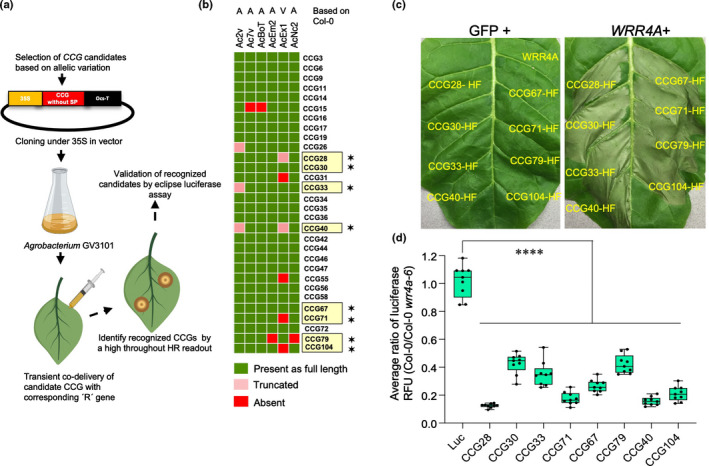
Identification of eight *CCG* genes from *Albugo candida* that elicit an hypersensitive response (HR) when co‐expressed in tobacco leaves with *WRR4A* from *Arabidopsis thaliana* accession Col‐0. (a) Pipeline for high‐throughput screening of the *CCG* genes for identification of recognised *CCG*s by *WRR4A* or *WRR4B*. Selected candidate effectors without the signal peptide (SP) were cloned into an expression vector under the CaMV‐35S promoter for *Agrobacterium*‐mediated transient expression in *Nicotiana tabacum*. Recognised *CCG*s were identified by HR phenotype and further validated by a luciferase eclipse assay. (b) Heatmap showing variation in *CCG* genes between *A. candida* races across 30 selected candidate *CCG* genes for co‐expression with *WRR4A*. Candidates were selected based on presence/absence across *A. candida* races and/or showing allelic variation in AcEx1, a Col‐0‐virulent isolate of race 4. The virulent (V) and avirulent (A) races on *WRR4A* are indicated based on their phenotype on Col‐0. The *WRR4A*‐recognised *CCG*s are highlighted with a yellow box and marked with a (✶). (c) Transient expression of *CCG* genes fused with a C‐terminal His‐FLAG (HF) tag upon co‐infiltration either with GFP or with *WRR4A* in *N. tabacum*. The eight identified *CCG*s trigger HR when co‐expressed with *WRR4A* but not with GFP alone. *WRR4A* does not cause HR when co‐delivered with GFP. (d) *WRR4A*‐dependent *CCG* recognition correlates with the reduction in luciferase activity upon particle bombardment into *A. thaliana* leaves of the recognised *CCG* with a luciferase construct compared with luciferase without *CCG* control. Graph shows the reduced luciferase activity of all recognised CCGs using a ratio of the measured luciferase in wild‐type Col‐0 compared with Col‐*wrr4a‐6* mutant, verifying that recognition is *WRR4A* dependent. Box plots show the median of the relative luciferase (Luc) activity from independent transfections, which are represented by dots and the box shows the interquartile range. Statistical significance versus Luc alone is indicated by asterisks (****, *P* < 0.0001) according to one‐way analysis of variance and Bonferroni's multiple comparison test.

**Fig. 3:**
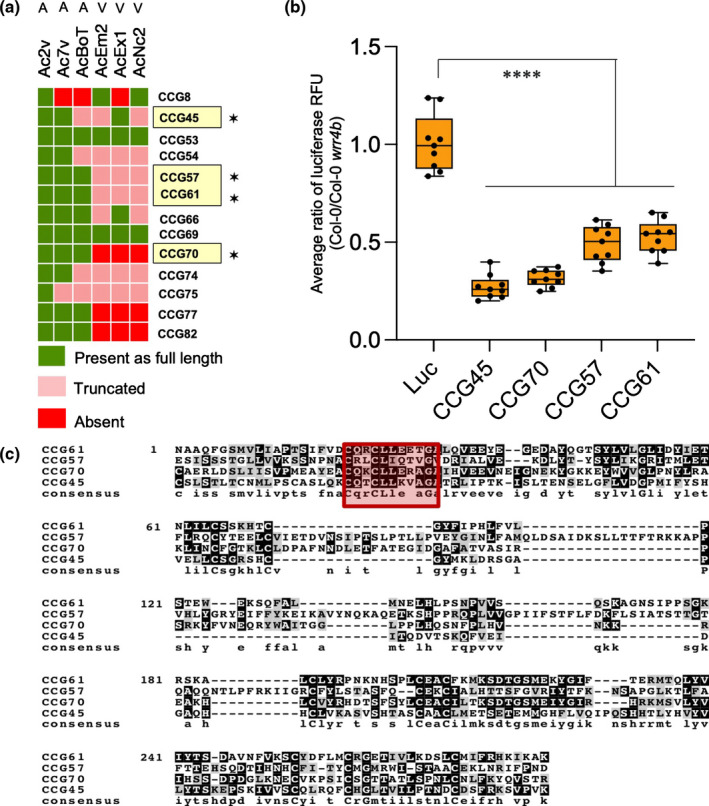
The *WRR4B* ^Col‐0^ paralogue recognises four additional CCG effectors of *Albugo candida*. (a) Heatmap showing variation in the *CCG* genes between different *A. candida* races across 13 candidate *WRR4B‐*recognised CCGs. Candidates were selected based on allelic polymorphism and presence/absence variation across different *A. candida* races infecting Brassica crops. Virulent (V) and avirulent (A) isolates on *Arabidopsis thaliana* accession Ws‐2 are indicated. The *WRR4B*‐recognised *CCG*s identified in our screen are marked with an asterisk (✶). (b) Luciferase eclipse assays upon particle bombardment in *A. thaliana*. Graph showing reduced luciferase activity of recognised *CCG*s identified by the ratio of luciferase activity in Col‐0 compared with the Col‐*wrr4b* mutant, verifying *WRR4B*‐dependent recognition. Box plots show the median of the relative luciferase (Luc) activity from independent transfections, which are represented by dots and the box shows the interquartile range. Statistical significance versus Luc alone is indicated by asterisks (****, *P* < 0.0001) according to one‐way analysis of variance and Bonferroni's multiple comparison test. (c) Multiple sequence alignment of *WRR4B*‐recognised CCGs that shows postsignal peptide homology primarily in the CCG motif. CCG motif is highlighted by a red box. All sequences shown in the figure are from isolate Ac2V that is resisted by *WRR4B*.

CCG candidates were transiently co‐expressed with *WRR4A* or *WRR4B* (Fig. 2a). Candidates selected to screen with *WRR4A* were mostly present across different races (indicated in green) and either absent or truncated in *WRR4A*‐overcoming race 4 isolate AcEx1 (indicated in red or pink) as shown in the heatmap (Fig. 2b). CCG candidates selected to screen with *WRR4B*, were mostly present in any or all of the *A. candida* races Ac2V, Ac7V and AcBoT that infect Brassica crops (indicated in green) but absent or truncated in other *A. candida* races as indicated in the heatmap showing presence/absence polymorphism (Fig. 3a). The different CCG variants that were tested for *WRR4*‐mediated recognition are listed in Table 2.

### *WRR4A* ^Col^ ^‐0^ confers recognition to eight different CCG effectors from *A. candida*

To screen candidate CCGs for their recognition by WRR4A, cloned effector alleles were co‐infiltrated with an *Agrobacterium* strain carrying 35S:*WRR4A*. Among the 30 alleles we tested, eight *CCG* genes (*CCG28* ^Ac2V^, *CCG30* ^AcNc2^, *CCG33* ^AcNc2^, *CCG40* ^AcEm2^, *CCG67* ^AcEm2^, *CCG71* ^AcNc2^, *CCG79* ^Ac2V^ and *CCG104* ^Ac2V^) elicited HR within 36–48 h post infiltration (hpi) when co‐expressed with *WRR4A* but not with GFP control (Fig. 2c). *CCG* genes that did not elicit HR when co‐expressed with *WRR4A* were defined as nonrecognised (Fig. S1). Expression of recognised and some nonrecognised effectors was verified by western blot (Fig. S1).

To validate the HR phenotypes, we carried out ‘luciferase eclipse’ assays using particle bombardment to reveal reduced luciferase activity upon HR triggered by these recognised *CCG* genes in Arabidopsis leaves in Col‐0 compared with Col‐0 *wrr4a‐6* mutant. All the *CCG* genes recognised by *WRR4A* conferred reduced luciferase activity at 48 h post bombardment in comparison with the luciferase‐alone control in Col‐0 but not in Col‐0 *wrr4a‐6* mutant. Diminished luciferase activity in Col‐0 indicates recognition‐dependent cell death in transformed leaf cells (Fig. 2d). Therefore, eight different CCGs are recognised by WRR4A.

### *WRR4B* ^Col‐0^ recognises four additional *CCG* genes

We tested if the *WRR4B* ^Col‐0^ paralogue at the *WRR4* locus in Arabidopsis also recognises specific *CCG* genes. As *WRR4B* confers resistance against *A. candida* race 2 (Ac2V), *CCG* genes from *A. candida* races primarily infecting Brassica crops and from Ac2V (Fig. 3a), were prioritised for co‐expression with *WRR4B*.

Transient expression of *WRR4B* in tobacco or *N. benthamiana* shows weak autoimmunity, even when infiltrated with GFP control (Fig. S2a,b), so any HR phenotypes detected upon co‐expression with a potentially recognised *CCG* gene must be cautiously interpreted. We used the luciferase eclipse assay for screening the selected set of *CCG*s for *WRR4B* recognition and discovered four *WRR4B‐*recognised *CCG* genes. We further confirmed the recognition of these four candidates (*CCG45* ^Ac2V^, *CCG57* ^Ac2V^, *CCG61* ^Ac2V^ and *CCG70* ^Ac2V^) with a luciferase eclipse assay by a comparison of the luciferase activity in Col‐0 and the Col‐0‐*wrr4b* mutant. Col‐0 *wrr4b* showed a higher level of luciferase when co‐expressed with these four *CCG* genes compared with Col‐0 (Fig. 3b), confirming *WRR4B*‐specific recognition of these *CCG* genes.

To test if any of these *CCG*s confer elevated HR after transient expression in *N. tabacum* compared with *WRR4B* alone, *CCG45* ^Ac2V^, *CCG57* ^Ac2V^, *CCG61* ^Ac2V^ and *CCG70* ^Ac2V^ were co‐infiltrated with *WRR4B*. Only *CCG45* ^Ac2V^ and *CCG70* ^Ac2V^ enhanced the HR compared with the *WRR4B* infiltrated with GFP alone, suggesting that they are recognised by *WRR4B* in tobacco (Fig. S2a). A modified HR index was used (Segretin *et al*., 2014) based on an arbitrary scale ranging from 0 (no visible necrosis) to 6 (full necrosis) (Fig. S2c,d). *CCG57* ^Ac2V^ and *CCG61* ^Ac2V^ did not elevate *WRR4B*‐dependent HR in *N. tabacum* compared with GFP control (Fig. S2a). We also co‐infiltrated *CCG45* ^Ac2V^ and *CCG70* ^Ac2V^ with *WRR4B* in *N. benthamiana*. *CCG28* ^Ac2V^ co‐delivered with *WRR4A* was used as a positive control. Consistent with the *N. tabacum* phenotype, *CCG45* ^Ac2V^ and *CCG70* ^Ac2V^ confer elevated HR in *N. benthamiana* when co‐expressed with *WRR4B* compared with *WRR4B* alone although the response varied between individual leaves (Fig. S2b,c). A protein alignment of these four *WRR4B*‐recognised CCGs shows that the CCG motif region is conserved (Fig. 3c). We conclude that WRR4B specifically recognises four CCGs from *A. candida* that are distinct from the CCGs recognised by WRR4A.

### Recognition of CCGs by WRR4A^Col^ ^‐0^ requires the N‐terminal part of the effector but does not occur via the CCG motif

**Fig. 4:**
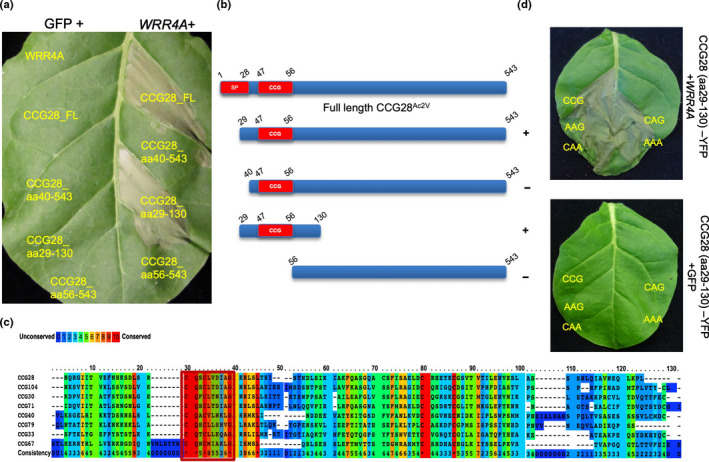
The N‐terminal postsignal peptide domain of CCG proteins is sufficient for recognition by WRR4A^Col‐0^ and recognition does not require the CCG motif. (a) Transient expression of full‐length (FL) postsignal peptide CCG28 and different truncated versions of CCG28 either with GFP or with WRR4A in *Nicotiana tabacum*. The N‐terminal region that corresponds to amino acids (aa) 29–130 is necessary and sufficient for WRR4A recognition. (b) Schematic representation of the FL CCG28, without the secretion signal and different N‐terminal versions of CCG28 infiltrated in Fig. 4a, showing either a hypersensitive response (HR) (+) or a No‐HR (−) phenotype. (c) The alignment of N‐terminal postsignal peptide CCG protein sequences up to amino acid 130 in all WRR4A‐recognised CCGs. The CCG motif is highlighted by a red box. Sequences shown in the figure are from isolates AcNc2 (CCG28, CCG30, CCG33, CCG71), AcEm2 (CCG40, CCG67) and Ac2V (CCG79, CCG104) that are resisted by *WRR4A*. (d) Mutations in the CCG motif with CCG changed to AAG, CAA, CAG and AAA do not impair WRR4A recognition and HR. Wild‐type or mutated variants of CCG28^aa29‐130^‐YFP were transiently co‐expressed in *N. tabacum* leaves with WRR4A, and all variants trigger HR at 48 h post infiltration (hpi). Photographs are representative of three replicates.

To map the region in the recognised CCG effectors that triggers HR, we focused on the WRR4A–CCG interaction, as WRR4A shows no autoimmunity. We selected CCG28 as it triggers the quickest *WRR4A*‐dependent HR, with an HR visible at 36 hpi. As the CCG motif region was the only part of the CCG protein that showed homology across different *WRR4A*‐recognised CCGs, we tested the role of the N‐terminal region of CCG28 for recognition by *WRR4A*. Several N‐terminal truncated versions of CCG28 of *c*. 100 amino acid residues after the secretion signal, surrounding and also including the CCG motif, trigger a fast HR upon transient co‐expression with *WRR4A*. However, deletion of amino acids 29–40 in the N‐terminal region after the secretion signal abolishes this HR (Fig. 4a,b).

To define the minimal N‐terminal region of CCG28 that is recognised, we made N‐terminal truncations of CCG28^Ac2V^ and tested their recognition using transient assays in *N. tabacum* leaves (Fig. S3a). A truncation of CCG28^Ac2V^ that includes the first 100 amino acids after the secretion signal site (CCG28^29‐130^), including the CCG motif, is recognised by *WRR4A* similarly to full‐length CCG28, triggering an HR at 36 hpi when transiently co‐expressed in *N. tabacum* (Figs 4a,b, S3a). By contrast, CCG28 without the CCG motif, corresponding to aa 56–543, does not trigger HR when co‐expressed with *WRR4A* (Figs 4a,b; S3b). Therefore, the N‐terminal part of CCG28, which corresponds to aa 29–130 after the signal peptide sequence, is indispensable for WRR4A recognition. CCG28^35‐130^ also fails to trigger recognition (Fig. S3a,c). To define the shortest region of CCG28 that is recognised, we further narrowed the N‐terminal part to 50 amino acids, corresponding to CCG28^29‐79^. However, only YFP‐tagged versions of this shortest region activate HR (Fig. S3a,c), probably indicating that untagged versions are insufficiently stable for their recognition by WRR4A to trigger a cell death phenotype when expressed transiently.

We tested whether recognition of all the WRR4A‐recognised CCG candidates involves their N‐terminal 100 amino acids. Upon transient expression, truncated versions of the other seven recognised CCGs trigger an HR phenotype when co‐expressed with *WRR4A* in *N. tabacum* (Fig. S4a). These data suggest that the N‐terminal part of these CCGs is sufficient for recognition by WRR4A. A mutation in the Walker A (P‐loop) of WRR4A (Schreiber *et al*., 2016) with a change from K220 > L220 abolishes the HR when *WRR4A* ^K220L^ is co‐infiltrated with recognised *CCG* genes (Fig. S4b). Prediction of protein structure of the 100 amino acid residues of CCG28, CCG30 and CCG71 with AlphaFold2 (Jumper *et al*., 2021) and their alignments indicate a structural similarity among these proteins (Fig. S5).

We next examined the role of the CCG motif in effector recognition (Fig. 4c) using the N‐terminal region of CCG28 corresponding to aa 29–130. Mutant versions of CCG28^29‐130^‐YFP carrying a mutation in the CCG motif (with CX_2_CX_5_G changed to AX_2_AX_5_G, CX_2_AX_5_A, CX_2_AX_5_G or AX_2_AX_5_A) were generated and tested for HR in transient assays after co‐infiltration with *WRR4A* in *N. tabacum*. These mutated versions are strongly recognised by WRR4A with an HR indistinguishable from CCG28^29‐130^‐YFP (Fig. 4d) suggesting that *WRR4A*‐CCG recognition does not occur via the CCG motif. Conceivably, a structural similarity in the N‐terminal part of these CCGs might underpin their detection by WRR4A. Consistent with this, CCG30, but not its close paralogue CCG16, is recognised by WRR4A (Fig. S6).

### *WRR4A* ‐recognised CCGs associate with WRR4A to trigger HR

**Fig. 5:**
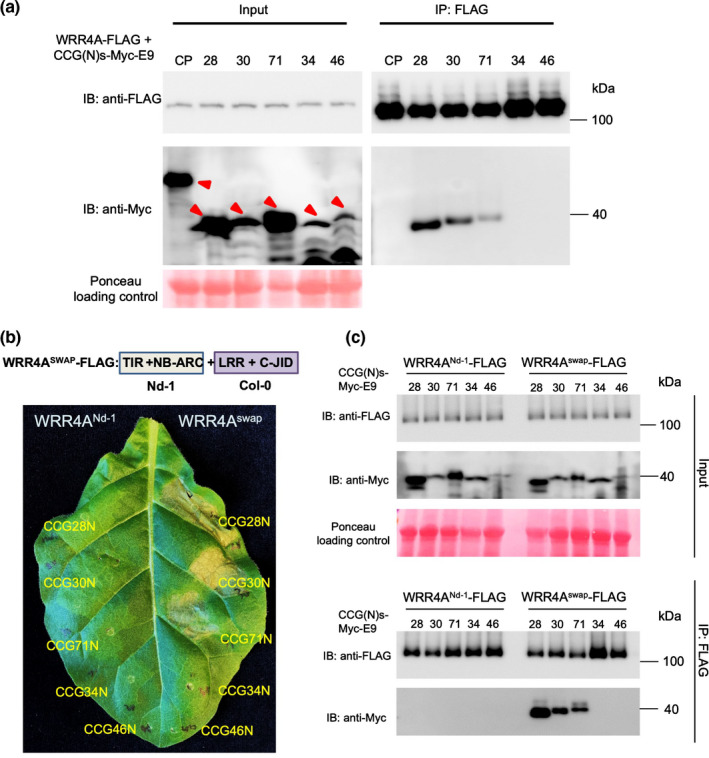
WRR4A recognises CCGs by interacting with their N‐terminal postsignal peptide domains. (a) WRR4A interacts with the N‐terminal region of recognised CCGs (CCG(N)) but not those of nonrecognised CCGs. FLAG epitope–WRR4A and recognised or nonrecognised Myc‐E9‐tagged CCG were transiently co‐expressed in *Nicotiana benthamiana* leaves. Protein extracts were immunoprecipitated with FLAG affinity gel and immunoblotted membranes analysed with anti‐FLAG and anti‐Myc antibodies. Red arrowheads indicate the predicted protein sizes of the Coat protein (CP) or the N‐terminal part of the corresponding CCGs tested for their interaction. (b) WRR4A^swap^ restores the recognition ability of the WRR4A^Nd‐1^ variant. N‐terminal regions of three WRR4A‐recognised and two WRR4A‐nonrecognised CCGs fused with Myc‐E9 tag were co‐infiltrated either with *WRR4A* ^Nd‐1^ or *WRR4A* ^swap^ in *N. tabacum*. WRR4A^Col‐0^‐recognised CCGs do not trigger a hypersensitive response (HR) when co‐expressed with *WRR4A* ^Nd‐1^. *WRR4A* ^swap^ carrying TIR and NB‐ARC from *WRR4A* ^Nd‐1^ and LRR and the C‐JID domain from *WRR4A* ^Col‐0^ confers recognition of WRR4A^Col‐0^‐recognised CCGs. (c) WRR4A^swap^ interacts with WRR4A^Col‐0^‐recognised CCGs*. N. benthamiana* leaves were harvested 3 d post infiltration (dpi). Protein extracts were immunoprecipitated with FLAG affinity gel and immunoblotted membranes analysed with anti‐FLAG and anti‐Myc antibodies. CP, coat protein of Potato virus Y (PVY) as the control. Results shown are representative of at least three independent replicates.

To investigate interaction of CCGs with WRR4A, we performed co‐immunoprecipitations (Co‐IPs) after transient expression of epitope‐tagged WRR4A and recognised CCG proteins in *N. benthamiana*. We tested CCG28, CCG30 and CCG71 (recognised by WRR4A) and CCG34 and CCG46 (nonrecognised by WRR4A) for interaction with WRR4A^Col‐0^. N‐terminal truncated versions of selected CCGs were generated with a C‐terminal Myc tag fused to *E. coli* colicin E9 for further purification due to its high affinity with Im9 (Kleanthous & Walker, 2001) and co‐expressed with WRR4A with a C‐terminal His‐FLAG (HF tag) via *Agrobacterium‐*mediated transformation. Only recognised CCGs associate with WRR4A (Fig. 5a). This suggests that interaction of recognised CCGs with WRR4A^Col‐0^ is necessary for defence activation.

To test whether the LRR and/or C‐JID domain of WRR4A^Col‐0^ mediate CCGs recognition, we tested the interaction of five CCG effectors (three recognised and two nonrecognised ones) with the nonfunctional WRR4A Niederzenz‐1 allele (*WRR4A* ^Nd‐1^) (Pucker *et al*., 2019), which differs by 22 amino acid residues from WRR4A^Col‐0^ (Fig. S7) and does not recognise the CCGs. Transient expression in *N. tabacum* and Co‐IP assays show WRR4A^Nd‐1^ does not interact with the N‐terminal versions of three WRR4A^Col‐0^‐recognised CCGs (CCG28‐N, CCG30‐N and CCG71‐N). A domain swap between WRR4A^Nd‐1^ and WRR4A^Col‐0^, which includes TIR and NB‐ARC of *WRR4A* ^Nd‐1^ and LRR and C‐JID domain of *WRR4A* ^Col‐0^ (WRR4A^swap^) recognises the three WRR4A^Col‐0^ recognised CCGs and also interacts in Co‐IP assays. However, the N‐terminal versions of two nonrecognised CCGs used in our assays do not interact with WRR4A^swap^ (Fig. 5b,c). This suggests that the WRR4A‐CCG recognition and interaction map to the LRR + C‐JID domain of WRR4A^Col‐0^ and recognition specificity resides in the LRR and/or C‐JID domain of WRR4A^Col‐0^, consistent with the hypothesis that direct CCG binding to WRR4A activates defence.

### Allelic variation and expression polymorphism of *WRR4A* recognised CCGs

**Fig. 6:**
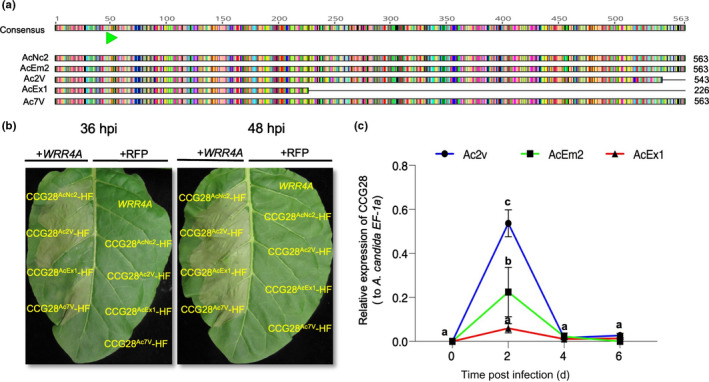
Differential recognition of CCG28 alleles from different *Albugo candida* races. (a) Schematic representation of the amino acid sequence alignment of CCG28 variants from *A. candida* race 2 (Ac2V), race7 (Ac7V) and race 4 (AcNc2, AcEm2 and AcEx1). Each amino acid residue is colour coded and similarity indicates conserved amino acid sequence across all races. Total protein length indicated as 563, 543 or 226 amino acids. The figure is drawn to scale. The CCG motif is indicated with a green arrow. (b) Recognition of different CCG28 variants from different *A. candida* isolates with a C‐terminally tagged His‐FLAG (HF) tag in a *Nicotiana tabacum* transient assay. The different CCG28 variants were co‐expressed with WRR4A or with RFP control in *N. tabacum* leaves. CCG28 shows the recognition with strong hypersensitive response (HR) at 36 hpi when co‐expressed with WRR4A. The truncated CCG28 from AcEx1, which overcomes the *WRR4A* recognition, shows a delayed HR at 48 hpi. (c) Expression analysis of CCG28 (relative to *A. candida EF‐1a*) in race 2 (Ac2v) and race 4 (AcEm2 and AcEx1), showing that the AcEx1 truncated allele of CCG28 is weakly expressed, consistent with virulence on Arabidopsis accessions with *WRR4A*. Different letters indicate statistically significant differences between the different alleles tested (two‐way analysis of variance and Bonferroni's multiple comparison test, *P* < 0.05). Error bars represent SD.

WRR4A‐recognised CCGs are present across different *A. candida* races, including AcEx1 that overcomes *WRR4A* resistance, so we investigated the AcEx1 alleles of *WRR4A*‐recognised *CCG*s. Several recognised *CCG* alleles show polymorphism in AcEx1 (Table S3). Due to an early stop codon, *CCG28* ^AcEx1^ encodes a protein of only 226 amino acids compared with full‐length alleles from other races (Fig. 6a). The AcEx1 *CCG28* allele apparently confers a delayed response in recognition compared with full‐length alleles from Ac2V, AcEm2 and Ac7V, all of that trigger an early *WRR4A*‐dependent HR at 36 hpi (Fig. 6b). Presence/absence polymorphism or truncation is also seen across some of the other *WRR4A‐*recognised *CCG*s. *CCG71* and *CCG104* are absent from AcEx1 (Fig. 2b; Table S3), consistent with absence of these effectors contributing to evasion of *WRR4A*‐mediated resistance. Moreover, *WRR4A*‐recognised *CCG33* and *CCG40* are truncated in race Ac2V (Fig. 2b; Table S3). Although AcEx1 can overcome *WRR4A* resistance, *WRR4A*‐recognised *CCG30*, *CCG33* and *CCG67* are present in AcEx1 and their encoded proteins are highly similar to the recognised AcEm2/Nc2 CCG variants (Notes S1). We investigated this discrepancy further.

Among *WRR4B‐*recognised *CCG*s in the *A. candida* races that primarily infect Brassica species, only *CCG45* is also present in AcEx1 (Table S4). *CCG45* ^AcEx1^ is highly diverged compared with *CCG45* ^Ac2V^ (Fig. S8a). To test if *CCG45* ^AcEx1^ also elevates HR compared with *WRR4B* alone in *N. benthamiana*, we co‐infiltrated these two alleles of *CCG45* with *WRR4B* in *N. benthamiana*. *CCG45* ^Ac2V^ triggers a more rapid HR in *N. benthamiana* when co‐expressed with *WRR4B* compared with *CCG45* ^AcEx1^ or *WRR4B* alone, although the response varied between individual leaves (Fig. S8b). Therefore, *CCG45* allelic variation could partially explain AcEx1 evasion of *WRR4B* recognition.

To test the expression of WRR4A‐ and WRR4B‐recognised CCGs *in planta*, we assessed RNA‐seq data obtained over a time course of infection by the *B. juncea* isolate Ac2V (Furzer *et al*., 2022). WRR4A‐ and WRR4B‐recognised *CCG* genes in Ac2V show *in planta* induction with expression peaks at different colonisation time points of 2, 4 and 6 dpi (Fig. S9a).

We investigated the expression of several alleles of *WRR4A*‐recognised *CCG* genes using qRT‐PCR expression profiling of Ac2V, AcEm2 and AcEx1 infections of Ws‐*eds1* plants (Fig. S9b). Different *WRR4A*‐recognised *CCG* genes are specifically expressed *in planta* during colonisation. Intriguingly, *CCG28* is primarily induced at 2 dpi. The Ac2V allele of *CCG28* shows the highest expression followed by the AcEm2 allele. The truncated allele from AcEx1 shows strikingly low expression compared with the other alleles (Fig. 6c), consistent with destabilisation of the early stop codon‐containing mRNA by nonsense‐mediated decay. The expression profiles of other *WRR4A*‐recognised *CCG*s such as *CCG30*, *CCG33* and *CCG67* that are present in all three *A. candida* races also show a similar trend in that, for each gene, the Ac2V allele is expressed significantly higher than the AcEm2 and AcEx1 alleles, which are expressed at similar levels (Fig. S9c). We infer that allelic variation, expression polymorphism, presence/absence variation in *CCG* genes and other unknown factors contribute to the growth of AcEx1 on *WRR4A*‐containing Col‐0.

**Fig. 7:**
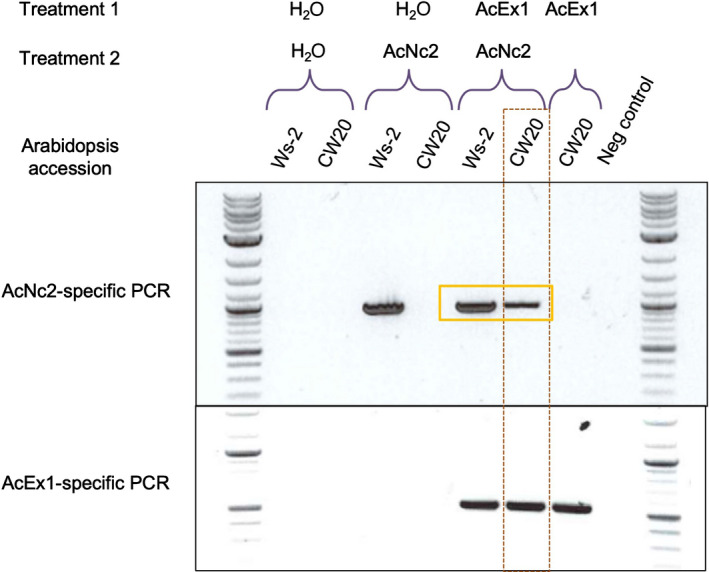
AcEx1 can suppress *WRR4A‐*mediated resistance. Co‐inoculation of AcEx1 and AcNc2 onto Arabidopsis accessions CW20 (*WRR4A*) and Ws‐2 (*wrr4a*) reveals that AcEx1 can suppress *WRR4*‐mediated immunity to AcNc2 (observed as AcNc2‐specific amplicon present in CW20 preinoculated with AcEx1). PCR amplification products highlighted in yellow box indicate the amplification of a secondary inoculum (AcNc2) on Ws‐2 control and *WRR4A*‐carrying CW20 upon primary inoculation with virulent *Albugo candida*(AcEx1). The red dotted box shows that suppression of WRR4A‐mediated resistance leads to growth of secondary *A. candida* infection.

*Albugo candida* infection immunocompromises hosts, enabling co‐infection by otherwise avirulent races, permitting sexual exchange and recombination (Cooper *et al*., 2008; McMullan *et al*., 2015). Although AcEx1 overcomes *WRR4A* resistance, it carries the *WRR4A*‐recognised *CCG30*, *CCG33* and *CCG67* genes and expresses them at a similar level to AcEm2 (Fig. S9). Conceivably, AcEx1 suppresses *WRR4A*‐mediated resistance. To assess this, we performed sequential inoculation of Arabidopsis accessions, and of recombinant inbred line (RIL) CW20, derived from a Col‐5 × Ws‐2 cross, which carries Col‐5 alleles of *WRR4A* and *WRR4B* but lacks other *WRR* genes. We monitored *A. candida* growth using race‐specific PCR amplicons. Isolate‐specific PCR verifies that AcNc2 can grow on Ws‐2 that does not have *WRR4A* but not on RIL CW20. However, CW20 plants colonised by race 4 isolate AcEx1 lose resistance to AcNc2 (Fig. 7). Therefore, AcEx1 not only suppresses *WRR4A*‐mediated recognition but also enables other races and even other nonadapted pathogens to grow, that would otherwise be resisted (Prince *et al*., 2017). These data suggest that AcEx1 is able to overcome *WRR4A* resistance not only by weak recognizability due to polymorphisms in its repertoire of *WRR4A*‐recognised *CCG* alleles, but also by immunosuppression of *WRR4A*‐mediated resistance.

### Some CCGs promote the growth of *Hpa in planta* and contribute to enhanced virulence

To test if any of the recognised or nonrecognised CCGs contribute to virulence function, we evaluated pathogen growth in *CCG*‐expressing transgenic lines of *A. thaliana*. We obtained homozygous transgenic plants that express one of five *CCG*s from recognised and nonrecognised CCG‐encoding genes (*CCG33*, *CCG44*, *CCG46*, *CCG47* and *CCG71*) and tested if their expression in *A. thaliana* Ws‐2 resulted in enhanced susceptibility to another oomycete pathogen *Hpa* (cause of downy mildew). Three out of five *CCG*‐expressing transgenic lines supported enhanced sporangiophore formation in *Hpa* Waco9 compared with nontransformed or *35S:GUS* expressing transgenic control plants (Fig. S10). However, this enhanced susceptibility was less than *Ws‐eds1*, which is hypersusceptible to pathogen growth. These data support the hypothesis that the secreted CCG proteins of *A. candida* act as effectors.

## Discussion

*Albugo candida* infection compromises Brassica yields, and infection renders hosts susceptible to secondary infections that would otherwise be resisted (Kamoun *et al*., 2015; Gupta *et al*., 2018). *Albugo candida* lacks secreted RxLR effector proteins encoded in the genomes of other phytopathogenic oomycetes such as *P. infestans* and *Hpa*, and the identity and nature of *Albugo* effectors were hitherto unknown. *A. candida* carries secreted CCG‐encoding genes that are located in the gene‐sparse and fast‐evolving part of the genome and show presence/absence variation and an elevated proportion of nonsynonymous to synonymous (pN/pS) changes, properties shared with effectors of other species (Furzer *et al*., 2022). We defined the CX_2_CX_5_G (‘CCG’) secretomes from different *A. candida* races and, using transient co‐expression in *Nicotiana* or Arabidopsis, we identified multiple CCG effectors recognised by two WRR4 paralogues. We also show that some of the identified CCG candidates promote susceptibility to another oomycete pathogen, *Hpa*, when expressed stably in Arabidopsis, further suggesting that secreted CCG proteins are part of the *Albugo* effector repertoire. This conclusion is consistent with our previous finding that *A. laibachii* CHxC proteins can enhance the virulence of *Pseudomonas syringae* DC3000 (Kemen *et al*., 2011).

Allelic comparisons enabled us to select CCG candidates based on their absence from *A. candida* races that overcome *WRR4* resistance. We identified eight WRR4A‐recognised CCGs and four WRR4B‐recognised CCGs. These *CCG* genes belong to clades that have specifically expanded in *A. candida* compared with *A. laibachii* (Furzer *et al*., 2022). The eight identified WRR4A‐recognised *CCG* genes when co‐expressed with *WRR4A* show an HR at 36–48 hpi. We verified their recognition by *WRR4A,* using luciferase eclipse assay after particle bombardment into wild‐type and a *wrr4a* mutant. Allelic comparison and transient assays revealed an additional four *CCG*s that are recognised by *WRR4B*. As *WRR4B* confers a weak autoimmune phenotype after transient expression in tobacco, co‐expression assays with effector candidates were difficult to interpret. Luciferase eclipse assays in Arabidopsis enabled us to identify four *WRR4B*‐recognised *CCGs*; *CCG45*, *CCG70*, *CCG57* and *CCG61*. *CCG45* and *CCG70* genes, which show the strongest ‘luciferase eclipse’ when bombarded into Col‐0 compared with Col‐0‐*wrr4b*, also show enhanced HR when co‐expressed transiently with *WRR4B* in *N. tabacum* or *N. benthamiana*. We conclude that WRR4A and WRR4B recognise multiple, distinct CCG effectors from *A. candida*. Multiple recognition of two effectors is known for some other NLRs that function in pairs, such as Arabidopsis RPS4/RRS1 (Sarris *et al*., 2015; Guo *et al*., 2020) and rice RGA4/RGA5 (Cesari *et al*., 2013). Moreover, singleton NLRs such as Arabidopsis RPM1 (Mackey *et al*., 2002) can recognise multiple unrelated effectors that target the RPM1‐guarded RIN4. Different alleles of the barley *Mla* immune receptor gene have evolved to detect sequence‐unrelated effectors from *Blumeria graminis* f. sp. *hordei* (*Bgh*) (Lu *et al*., 2016; Saur *et al*., 2019). Similarly, five sequence‐unrelated AvrPm3 effector proteins from wheat powdery mildew, which have a high structural similarity, are perceived by three alleles of the wheat NLR receptor *PM3* (Bourras *et al*., 2019). Our CCGs–WRR4A recognition data reveal an extreme example of how the same singleton NLR allele can detect multiple sequence‐unrelated effectors from different *A. candida* races, most likely to be directly. WRR4B can also recognise multiple CCG effectors.

For multiple CCGs, the N‐terminal 100 amino acids after the secretion signal are sufficient for recognition by WRR4A. Truncations of the WRR4A‐recognised CCG28 show that the N‐terminal 12 amino acid residues after the signal peptide cleavage site are required. All tested WRR4A‐recognised CCGs trigger immune activation with their N‐terminal 100 amino acids, and the C‐terminal portion of the CCG is dispensable for recognizability. Mutants of recognised CCGs in which CX_2_CX_5_G is mutated to AX_2_AX_5_A are still recognised by WRR4A, and the function of the CCG motif remains unknown. CCG proteins fall into different clades in a phylogeny built around the 60 amino acids surrounding and including the CCG motif (Furzer *et al*., 2022). A structural prediction of the N‐terminal part of WRR4A‐recognised CCGs using AlphaFold2 suggests conformational similarities between them (Jumper *et al*., 2021). Absence of recognition of CCGs by WRR4A^Col‐0^ might be caused by amino acid differences in the interaction interface, rather than large structural differences. Consistent with this, CCG16, a close paralogue of the WRR4A‐recognised CCG30 is not recognised despite a higher homology to CCG30 than other WRR4A‐recognised CCGs. Previous studies on tomato and rice NLRs *Xo1* and *Bs4* have shown that the recognition of closely related TAL effectors can involve their structural similarity rather than their transcriptional activity (Schornack *et al*., 2006; Triplett *et al*., 2016). Also, a recent study on recognition by the TIR–NLR Ry_sto_ showed that structurally conserved features in the coat protein of multiple potyviruses trigger its activation (Grech‐Baran *et al*., 2022).

Does WRR4A recognise distinct CCG effectors directly? WRR4A co‐immunoprecipitates only with recognised CCGs. Moreover, transient expression and a comparative Co‐IP analysis of WRR4A^Col‐0^ and WRR4A^Nd‐1^, which differ only by 22 amino acid residues, revealed the domains of WRR4A^Col‐0^ that mediate CCG detection. Transient expression and Co‐IP assays show that WRR4A^Nd‐1^ does not interact with the N‐terminal part of three different *WRR4A* ^Col‐0^‐recognised CCGs (CCG28‐N, CCG30‐N and CCG71‐N). Domain swaps between *WRR4A* ^Nd‐1^ and *WRR4A* ^Col‐0^ show that the TIR and NB‐ARC of WRR4A^Nd1^ are functional and that the recognition specificity resides in the LRR and/or C‐JID domains, consistent with RPP1/ATR1 and ROQ1/XopQ interactions (Ma *et al*., 2020; Martin *et al*., 2020). The WRR4A^Nd‐1^ and WRR4A^Col‐0^ LRR and C‐JID domains differ by only seven amino acids. Domain swap experiments for RPP1 variants for ATR1 recognition (Krasileva *et al*., 2010) and flax L6 variants for AvrL567 recognition (Ravensdale *et al*., 2012) gave similar results, consistent with direct interaction. WRR4B also has a post‐LRR C‐JID domain. As the WRR4A–CCG interaction maps to the LRR + C‐JID domain, by analogy with RPP1/ATR1 and Roq1/XopQ interactions, we propose that the most parsimonious explanation is that direct recognition underpins the detection of and response to CCGs by WRR4A, although other more complex models cannot be excluded.

The *A. candida* race 4 isolate AcEx1 overcomes *WRR4A‐* and *WRR4B*‐mediated resistance, and colonises Col‐0. This is partly explained by the loss of or allelic variation in some of the recognised AcEx1 effectors, and reduced expression of others. Additionally, AcEx1 suppresses *WRR4*‐mediated immunity against *A. candida* AcNc2. We propose that the partial susceptibility of Col‐0 plants to AcEx1 is explained not only by allelic variation and reduced expression of some of the *WRR4A*‐recognised *CCG* genes, resulting in weak or delayed recognition of otherwise strongly detected CCG effectors, but also by immunosuppression of this weak recognition.

Some accessions carry *WRR4A* alleles that confer resistance to AcEx1 (Fairhead, 2016; Castel *et al*., 2021). HR‐5 and Oy‐0 *WRR4A* alleles carry an extended C‐terminus that confers AcEx1 resistance by recognising additional CCG effectors. The identification of *WRR4A‐* and *WRR4B‐*recognised *CCG* genes will further advance the understanding of the recognition mechanism at the structural level. Therefore, this work contributes new insights into effector biology in obligate biotrophs, and will help to provide durable resistance in Brassicaceae crops by pyramiding or transgene stacking of different *WRR4* paralogues, which recognise diverse repertoires of *CCG* effector genes.

## Competing interests

None declared.

## Author contributions

AR, VC and JDGJ conceptualised and designed the research. AR, VC, KB, HZ, DSK, ZZ and SF conducted all experiments. AR, VC, KB, HZ, DSK, OJF and SF performed the data analysis. MHB and EBH gave critical intellectual input and provided material for this work. AR, VC and JDGJ wrote the manuscript with input from all co‐authors. AR and VC contributed equally to this work.

## Supporting information


**Fig. S1** Confirmation of expression of WRR4A‐recognised and representative nonrecognised CCGs.
**Fig. S2** *WRR4B* shows an enhanced hypersensitive responses with CCG45^Ac2V^ and CCG70^Ac2V^.
**Fig. S3** CCG28 recognition requires the N‐terminal 100 amino acids postsignal peptide.
**Fig. S4** CCG N‐terminal part is sufficient for recognition by WRR4A and requires an intact P‐loop in *WRR4A*.
**Fig. S5** Computational structural prediction of the CCG N‐terminal part of WRR4A‐recognised CCGs reveals structural similarity.
**Fig. S6** WRR4A recognises N‐terminal region of CCG30 but not close paralogue CCG16.
**Fig. S7** Sequence alignment between WRR4A^Col‐0^ and WRR4A^Nd‐1^.
**Fig. S8** CCG45^Ex1^ does not show enhanced *WRR4B*‐dependent hypersensitive responses compared with CCG45^Ac2V^.
**Fig. S9** Expression profiling of recognised *CCGs* by RNA‐seq and reverse transcription quantitative PCR analysis.
**Fig. S10** Some CCG effectors elevate susceptibility to *Hyaloperonospora arabidopsidis* Waco9 when constitutively expressed *in planta*.

**Notes S1** Sequence variation in all the recognised CCGs from different *Albugo candida* isolates.

**Table S1** Oligonucleotides used in this study.

**Table S2** Nucleotide and protein sequences of all the candidate CCGs tested in this study.

**Table S3** Allelic status and presence/absence variation of the WRR4A‐recognised CCGs across different *Albugo candida* isolates.
**Table S4** Allelic status and presence/absence variation of the WRR4B‐recognised CCGs across different *Albugo candida* isolates.
Please note: Wiley Blackwell are not responsible for the content or functionality of any Supporting Information supplied by the authors. Any queries (other than missing material) should be directed to the *New Phytologist* Central Office.

## Data Availability

The sequence data for all the CCGs reported in this study are openly available in NCBI and all accession numbers of the individual CCGs are given in Table S2.
