# Supplementary material for: The Arabidopsis WRR4A and WRR4B paralogous NLR proteins both confer recognition of multiple Albugo candida effectors
Source: New Phytol. 2022 Aug 7;237(2):532–47. doi: 10.1111/nph.18378 (PMC10087428; doi:10.1111/nph.18378)
Supplement: Supplementary file 1 — Fig. S1 Confirmation of expression of WRR4A‐recognised and representative nonrecognised CCGs. Fig. S2 WRR4B shows an enhanced hypersensitive responses with CCG45Ac2V and CCG70Ac2V. Fig. S3 CCG28 recognition requires the N‐terminal 100 amino acids postsignal peptide. Fig. S4 CCG N‐terminal part is sufficient for recognition by WRR4A and requires an intact P‐loop in WRR4A. Fig. S5 Computational structural prediction of the CCG N‐terminal part of WRR4A‐recognised CCGs reveals structural similarity. Fig. S6 WRR4A recognises N‐terminal region of CCG30 but not close paralogue CCG16. Fig. S7 Sequence alignment between WRR4ACol‐0 and WRR4ANd‐1. Fig. S8 CCG45Ex1 does not show enhanced WRR4B‐dependent hypersensitive responses compared with CCG45Ac2V. Fig. S9 Expression profiling of recognised CCGs by RNA‐seq and reverse transcription quantitative PCR analysis. Fig. S10 Some CCG effectors elevate susceptibility to Hyaloperonospora arabidopsidis Waco9 when constitutively expressed in planta. [file NPH-237-532-s005.pdf]

New Phytologist Supporting Information

Article title: **The Arabidopsis *WRR4A* and *WRR4B* paralogous NLR proteins both confer recognition of multiple *Albugo candida* effectors**

Authors: Amey Redkar, Volkan Cevik, Kate Bailey, He Zhao, Dae Sung Kim, Zhou Zou, Oliver J. Furzer, Sebastian Fairhead, M. Hossein Borhan, Eric B. Holub and Jonathan D.G. Jones

Article acceptance date: 5 July 2022

The following Supporting Information is available for this article:

Fig. S1 Confirmation of expression of WRR4A-recognized and representative non-recognized CCGs.

Fig. S2 *WRR4B* shows an enhanced HR with CCG45^{Ac2V} and CCG70^{Ac2V}.

Fig. S3 CCG28 recognition requires the N-terminal part 100 amino acids post-signal peptide.

Fig. S4 CCG N-terminal part is sufficient for recognition by WRR4A and requires an intact P-loop in *WRR4A*.

Fig. S5 Computational structural prediction of the CCG N-terminal part of WRR4A-recognized CCGs reveals structural similarity.

Fig. S6 WRR4A recognizes N-Terminal region of CCG30 but not close paralog CCG16

Fig. S7 Sequence alignment between WRR4A^{Col-0} and WRR4A^{Nd-1}.

Fig. S8 CCG45^{Ex1} does not show enhanced *WRR4B*-dependent HR compared to CCG45^{Ac2V}.

Fig. S9 Expression profiling of recognized *CCGs* by RNASeq and RT-qPCR analysis.

Fig. S10 Some CCG effectors elevate susceptibility to *H. arabidopsidis (Hpa)* Waco9 when constitutively expressed *in planta.*

Table S1 Oligonucleotides used in this study (see separate file).

Table S2 Nucleotide and Protein sequences of all the candidate CCGs tested in this study (see separate file).

Table S3 Allelic status and presence/absence variation of the WRR4A-recognized CCGs across different *A. candida* isolates (see separate file).

Table S4 Allelic status and presence/absence variation of the WRR4B-recognized CCGs across different *A. candida* isolates (see separate file).

Notes S1 Sequence variation in all the recognized CCGs from different *A. candida* isolates (see separate file).

Fig. S1

(a)

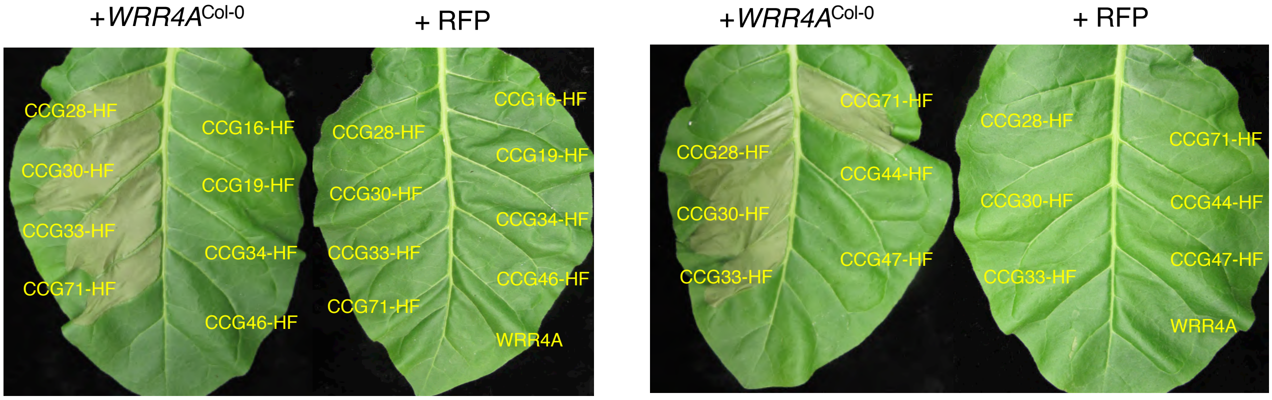

(b)

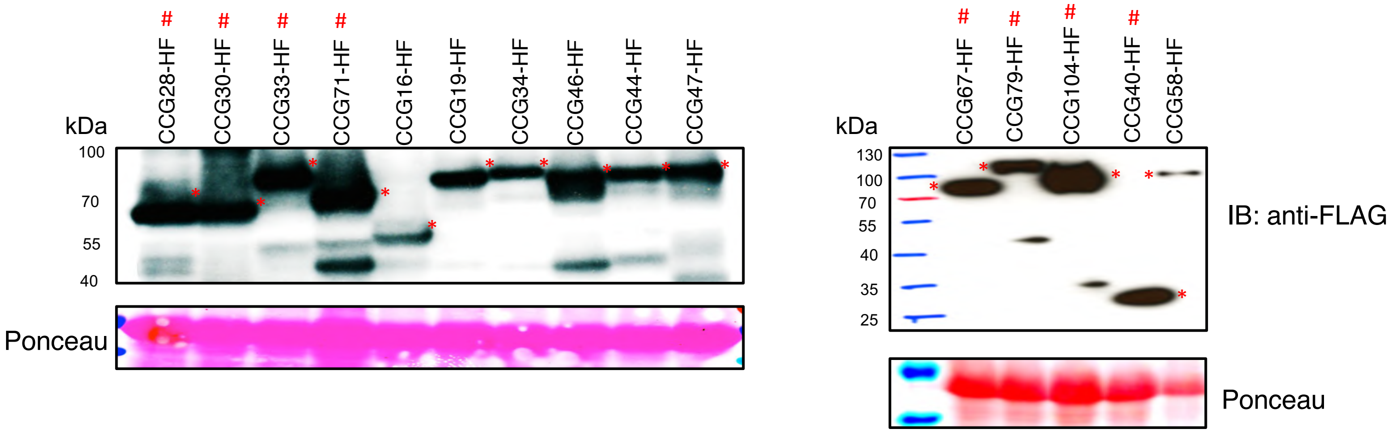

Fig. S1 Confirmation of expression of WRR4A-recognized and representative non-recognized CCGs.
(a) Transient expression of candidate recognized CCGs fused with a C-terminal His-FLAG (HF) tag upon co-infiltration either with RFP or with *WRR4A* in *N. tabacum*. Only the WRR4A-recognized CCGs trigger HR when co-expressed with *WRR4A* but not non-recognized CCGs. The recognized or non-recognized CCGs do not confer HR when co-delivered with RFP.
(b) Western Blot analysis of C-terminally His-FLAG (HF) tagged recognized and non-recognized CCGs. The CCGs were expressed under 35S promoter in *N. benthamiana* and leaf samples were taken 3 days post infiltration (dpi). Asterisk indicates expected protein size. Loading control is shown by a ´Ponceau-stained´ gel. The recognized CCGs are indicated by a #.

Fig. S2

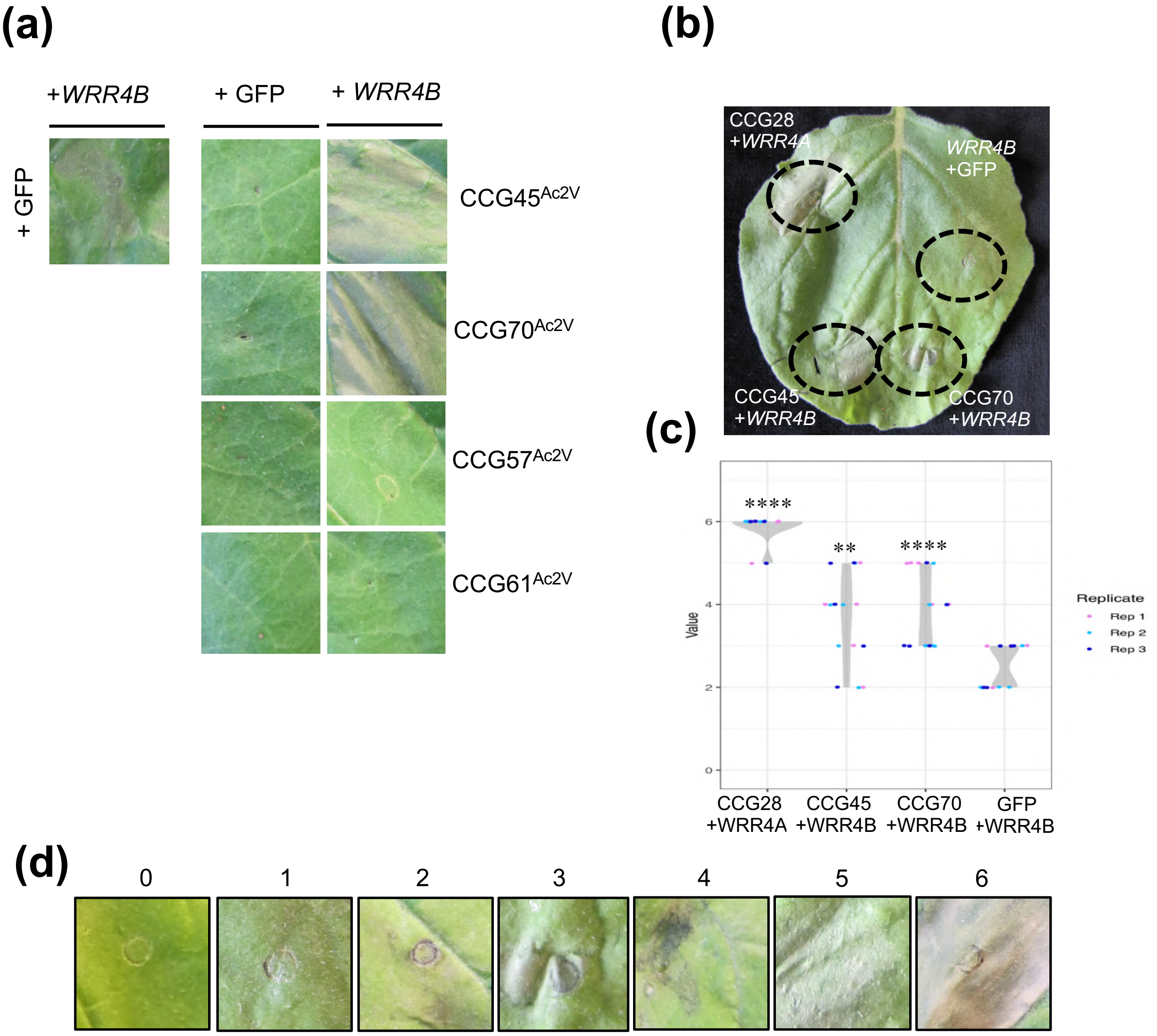

Fig. S2 *WRR4B* shows an enhanced HR with CCG45^{Ac2V} and CCG70^{Ac2V}

(a) *WRR4B* confers enhanced HR following co-expression with *CCG45*^{Ac2V} and *CCG70*^{Ac2V} in *N. tabacum.* Transient expression of candidate CCG effectors either with GFP or with *WRR4B* in *N. tabacum*. *WRR4B* triggers an enhanced HR when co-expressed with *CCG45*^{Ac2V} and *CCG70*^{Ac2V} compared to a weaker autoimmune phenotype when co-expressed with GFP. CCG57^{Ac2V} and CCG61^{Ac2V} do not show a stronger HR compared to GFP.

(b) No cell death observed in *N. benthamiana* leaves after expression of candidates CCG45^{Ac2V} and CCG70^{Ac2V}. *CCG28*^{Ac2V} co-infiltrated with *WRR4A* was used as a positive control. *N. benthamiana* leaf panels were photographed four days after *Agrobacterium* infiltration.

(c) Violin plots showing cell death intensity scored as an HR index based on three independent experiments. Statistical significance versus GFP +WRR4B alone. Asterisk indicate statistical significance (one-way ANOVA, Bonferroni´s multiple comparison test, **,P<0.01; ****,P<0.0001).

(d) HR index used for scoring macroscopic cell death phenotypes based on an arbitrary scale ranging from 0 (no visible necrosis) to 6 (full necrosis) as modified from Segretin *et al.,* 2014.

Fig. S3

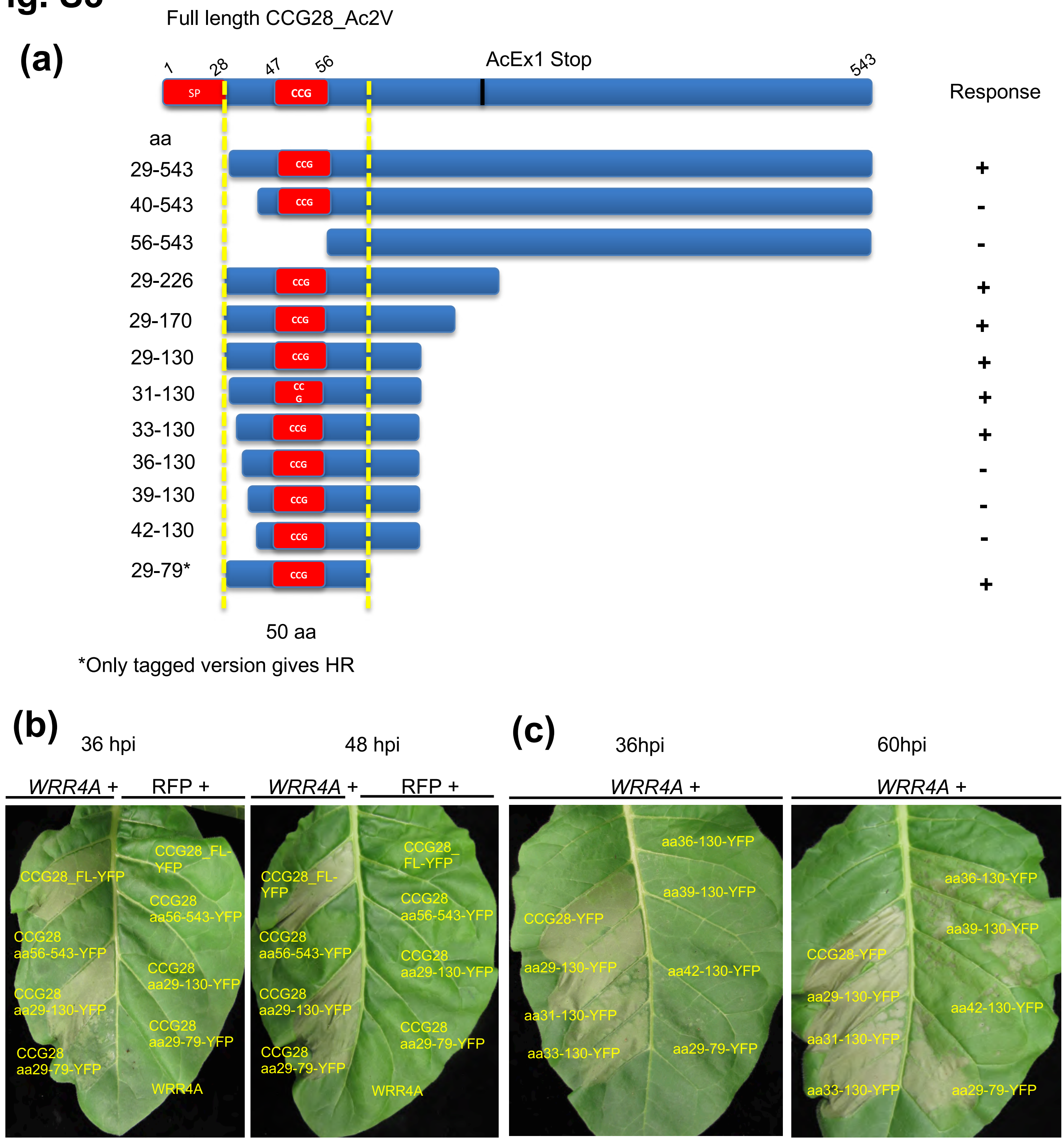
Full length CCG28_Ac2V
(a)
1
28
47
56
AcEx1 Stop
543
SP
CCG
Response
aa
29-543 +
40-543 -
56-543 -
29-226 +
29-170 +
29-130 +
31-130 +
33-130 +
36-130 -
39-130 -
42-130 -
29-79* +
50 aa
*Only tagged version gives HR
(b)
36 hpi
48 hpi
WRR4A +
RFP +
CCG28_FL-YFP
CCG28 aa56-543-YFP
CCG28 aa29-130-YFP
CCG28 aa29-79-YFP
WRR4A
(c)
36hpi
60hpi
WRR4A +
CCG28-YFP
aa29-130-YFP
aa31-130-YFP
aa33-130-YFP
aa36-130-YFP
aa39-130-YFP
aa42-130-YFP
aa29-79-YFP

Fig. S3 CCG28 recognition requires the N-terminal 100 amino acids post-signal peptide

(a) Cartoon illustrating various CCG28 truncations tested to define the minimal N-terminal region that recognized by WRR4A upon transient co-infiltration in *N. tabacum*. The + or - HR phenotype in each test is indicated. aa refers to the amino acids

(b) Transient expression of different truncations of CCG28 either with RFP or with *WRR4A* in *N. tabacum*. A truncation of CCG28 that includes the 100 amino acids post-signal peptide site ($CCG28^{29-130}$), including the CCG motif, is sufficient for recognition by WRR4A when transiently co-expressed in *N. tabacum.* Moreover, a further deletion narrowing the recognition region to 50 amino acids, corresponding to $CCG28^{29-79}$ is also recognized. However, only YFP-tagged versions of this shortest region activate HR. In contrast, a C-terminal region of CCG28 without the CCG motif, which corresponds to aa 56-543 abolishes recognition when co-expressed with *WRR4A*.

(c) Amino acids 29-33 of CCG28 are essential for early recognition at 36 hours post infiltration (hpi). Truncated versions of CCG28 that do not have aa 29-33 are delayed in recognition and weakly recognized 60 hpi.

Fig. S4

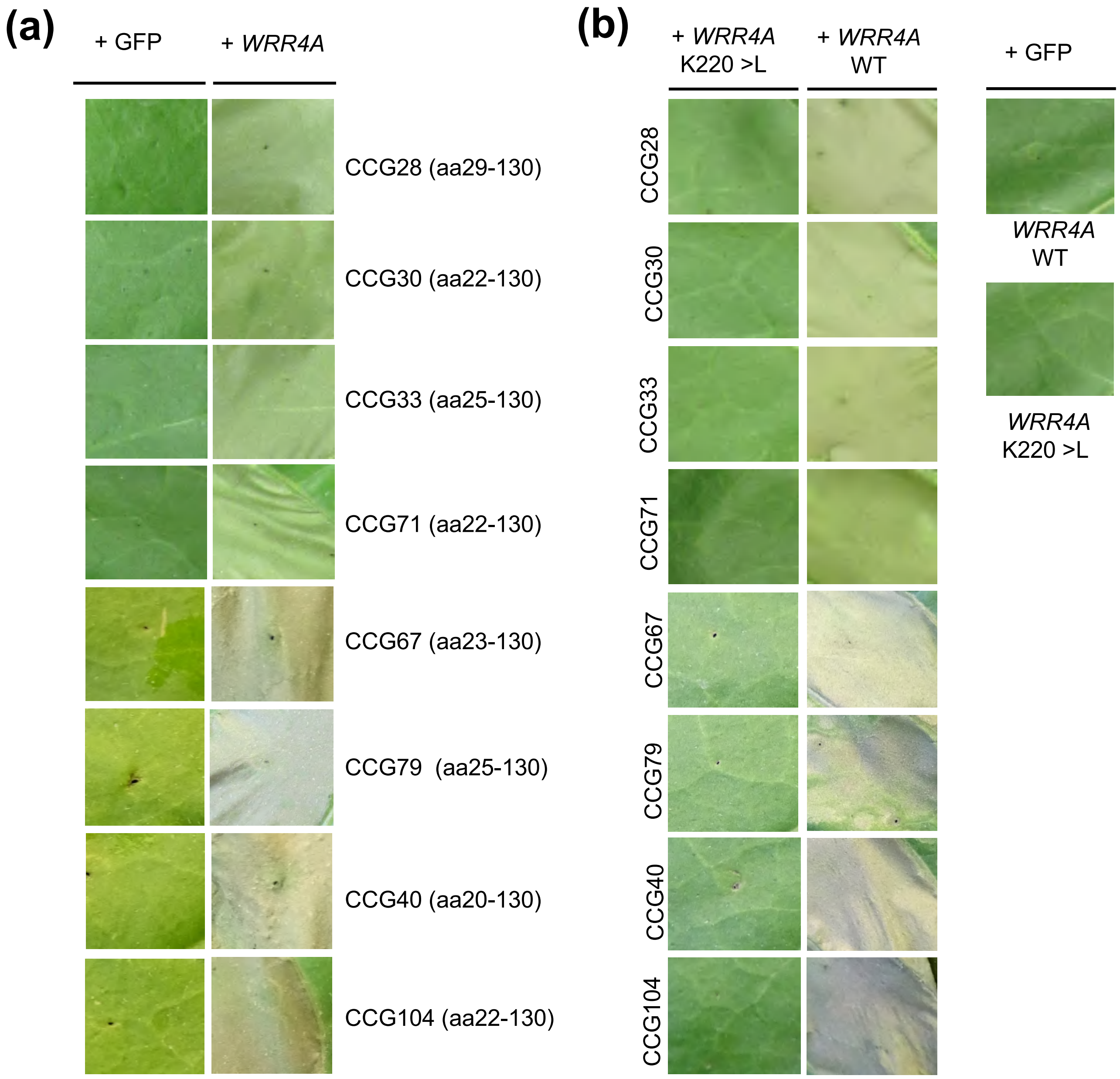

Fig. S4 CCG N-terminal part is sufficient for recognition by WRR4A and requires an intact P-loop in *WRR4A*

(a) Transient co-expression of different truncated versions of all WRR4A-recognized CCGs either with GFP or with WRR4A in *N. tabacum*. The N-terminal region of all recognized CCGs is sufficient for WRR4A recognition.

(b) *WRR4A*-mediated CCG recognition is dependent on intact Walker A (P-loop) motif.

Fig. S5

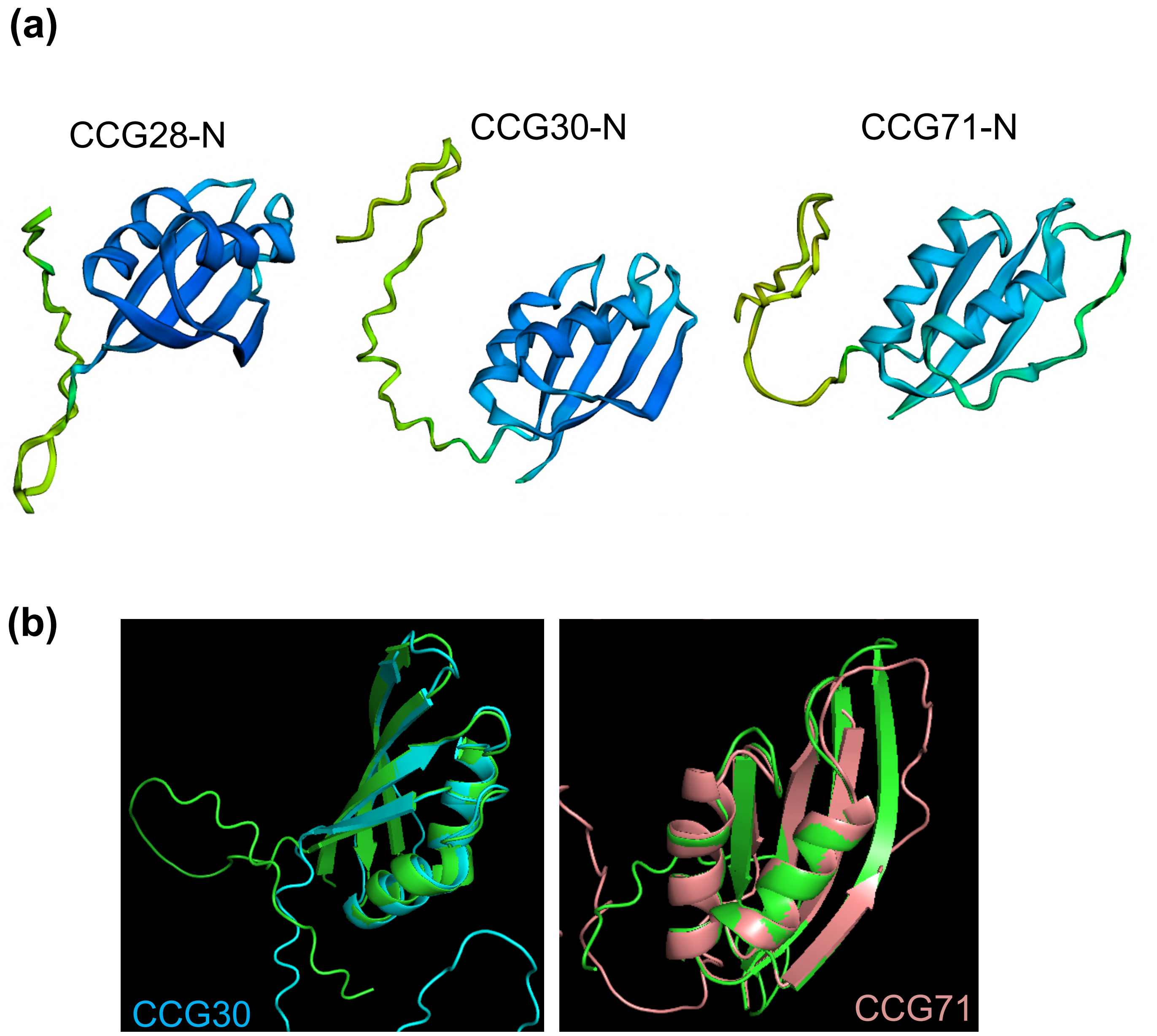

Fig. S5 Computational structural prediction of the CCG N-terminal part of WRR4A-recognized CCGs reveals structural similarity

(a) AlphaFold2 predicted protein structure of the N-terminal 100 amino acid region of different WRR4A-recognized CCGs (CCG28N, CCG30N and CCG71N).

(b) A structural alignment of the CCG N-terminal 100 amino acid region of CCG28 superimposed with that of N-terminal 100 amino acid (aa) region of CCG30 and CCG71. CCG28 N-terminal part is indicated in green.

Fig. S6

▼ Recognised region of CCG30

```
                                        ▼
CCG30_AcNc2    MIHPYWLFLSVVCLRNKVTNAKDPVIITATFSTDSNLGECQFCLTNIAGVNRISLLSITT    60
CCG16_AcNc2    MIHPYWLFLSVVCLRNAVTDAKDPVIITATFSVDSNLGECQFCLTNIAGVDRISLGSIST    60
               **************** **:************.*****************:**** **:*

CCG30_AcNc2    HYSNPPSATAILEAFGLVSSFLRAGIECQGKSKCGSITMTGLEHVNIKPSLETAKKPRCV    120
CCG16_AcNc2    YYSNPPSATAILEAYGWVSSFQRAGIECQGKSKCGFVTMTGLEHVDIDSSLRNMNAPLCV    120
               :*************:* **** ************* :********:*. **.. : * **

                         ▼
CCG30_AcNc2    LTDVQTTFECGVCLQMASGSHMIEKFLLVTDDETKHYFHVLYTTQSNIFEPCSFSGICSG    180
CCG16_AcNc2    LTDVQTTFECGICLQMVSGSHVIEKKQLVTHDRSEHYLHVLYTMQRDIFEPCSFSGICSG    180
               ***********:****.****:***  ***.*.::**:***** * :*************

CCG30_AcNc2    VSYEFADNTCVNLVNKLNQFHFRSVQVAPSSEPKPNLAKMHVAIKAVYDTTLSPSSCFKS    240
CCG16_AcNc2    VNYEFDDSTCVNLINKLTKYHFSSVQVDHLDRPKPKPAKMRVAIKAIDHTTQRTSLCYNT    240
               *.*** *.*****:***.::** ****    ..***: ***:*****: .**   * *:::
```

Fig. S6 WRR4A recognizes N-Terminal region of CCG30 but not close paralog CCG16
Alignment of protein sequences of the two paralogs CCG30 and CCG16 which show high identity in their sequence but CCG16 is not recognized by *WRR4A*. The red triangles indicate the minimal recognized region for CCG30. The consensus symbols in the alignment indicate the following; An * (asterisk) indicates positions which have a single, fully conserved residue. A : (colon) indicates conservation between groups of strongly similar properties - scoring > 0.5 in the Gonnet PAM 250 matrix. A . (period) indicates conservation between groups of weakly similar properties - scoring =< 0.5 in the Gonnet PAM 250 matrix.

Fig. S7

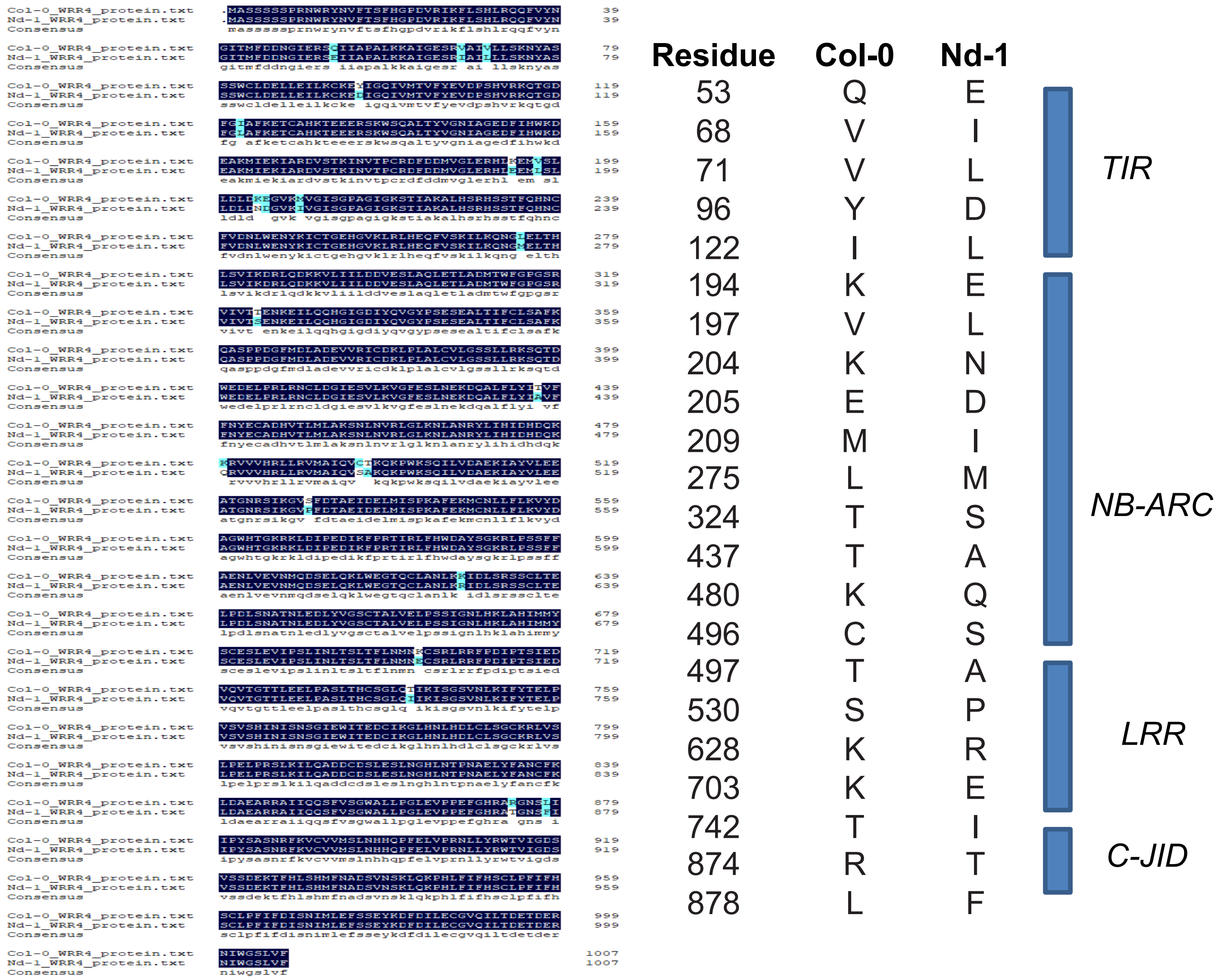

Fig. S7 Sequence alignment between WRR4A$^{Col\text{-}0}$ and WRR4A$^{Nd\text{-}1}$

Sequence alignment between WRR4A$^{Col\text{-}0}$ and WRR4A$^{Nd\text{-}1}$ which differ in 22 amino acid residues. WRR4A$^{Nd\text{-}1}$ does not recognize any CCG effectors. Differences in amino acid residues and their positions are highlighted.

Fig. S8

(a)

```
CCG45_Ex1     1   MEKSLVLVFLVIYASIYITECSLSILRYTIYQSHASLQTCQTCLLEVAGATRLIPMKNSPTENSELVFLVDGPMIFYSHV  80
CCG45_Ac2V    1   MEKSLALVFLVINASIYMTECSLSTLTCNMLPSCASLQKCQTCLLKVAGATRLIPTKISLTENSELGFLVDGPMIFYSHV  80

CCG45_Ex1     81  ELLCSKRSLCGYMKLDRSGAPITQDVTSKQFMEKDGAQYHCLMKTSVSHTASCAACLMETSETEMMGHFLVHVPQ-RFTL  159
CCG45_Ac2V    81  ELLCSGRSHCGYMKLDRSGAPITQDVTSKQFVEIDGAQHHCLVKASVSHTASCAACLMETSETEMMGHFLVQIPQSHHTL  160

CCG45_Ex1     160 HHMYILYTSKEPSKIVVSCQSRQFCHGLTVILPTNDCDLFRKSVPVKGMNRLPLESSGGHRLGEKVALYDTDTRNNAAQS  239
CCG45_Ac2V    161 YHVYVLYTSKEPSKIVVSCQLRQFCHGLTVILPTNDCDSFRKSVPVKGMNRLPLESYGSHRLGEKVALYDTDTRSDAAQL  240

CCG45_Ex1     240 FVSAIKQLTTMDRYCFNASSVEKNKEFCTTCMLNGKNRIILAVRTATGVDQVEASEAHKKSSVQHLFCLFTNEKVDWENL  319
CCG45_Ac2V    241 FVDAIKQLTTVDRYCFHASSVRNNTKFCTTCMLSEKDRIILAVRTAT-VDQVEASEANKKSSVEHLFCLFTNEKVDWENL  319

CCG45_Ex1     320 KSLCGGSKDSEPDPHRSTGKKKSRVSPCGTITPVGSGDDYSVPFDSQFYSFINKPVVKRLGNQISNAELCFWVQEERKQK  399
CCG45_Ac2V    320 KSVCGDSKDSEPDRHRAPEKQQPRVSSCGTITPVGSGNDYFVPFDSQFYSFINKPIVKRLGNQIRNAELCFWVQEERKQK  399

CCG45_Ex1     400 EQPNCLDCLFTSGMKLELSLSAALLTTTKSKVLDCLEDHSQCHPLRDVPIAMCQYEKELQLLALEESKRDVIPDLDLNKE  479
CCG45_Ac2V    400 EQPDCLNCLFKQGMKLELSLSAALLTITNSKVLDCVEDHSQCHPLRDVPIAMCRYEKELQLLALEESKMDVIPDLDLNKE  479

CCG45_Ex1     480 PHPDRHTFALKESKRDVIPDLDLNK--------------------------EPPPDRHPSRVETERTEKTTSYFTGTTPL  533
CCG45_Ac2V    480 PPPDRHTFALKKSKRDVIPDLDLNKEPHPDRHTLTLEESTRDVIPELDLNREPPPDRHSSRVE---PEKTTSYFTGTTPL  556

CCG45_Ex1     534 VIHFYLDEEIELFKCYECVLLYKEVLLMSVKRAYLWITNSAKGMNDLSSCSEHCRIKQVPSQNQFYFENFERLQPLSIND  613
CCG45_Ac2V    557 VIHFNLDEEIELFKCYECVLRYKEVLLMSVKRAYLWITNNAKGINHLSLCSKLCPIKHVPLQNQFSFENFERLQPLSIKD  636

CCG45_Ex1     614 VVFIFCGHIALSKRDKTSGASKRVGKDKKLTTSKDGLKTAKYYKSCISSLESRSRKIANSNEKRVRPVDKIWEFPTWTIR  693
CCG45_Ac2V    637 VVFIFCGDIPLLTRDKASGASKRVGKH---IMPKDRFKTTKYYKSCISSLESRSRKIANSNEKRVRPAEKNWEFPIWTIR  713

CCG45_Ex1     694 KKRKKRQ  700
CCG45_Ac2V    714 KKRKKNE  720
```

(b)

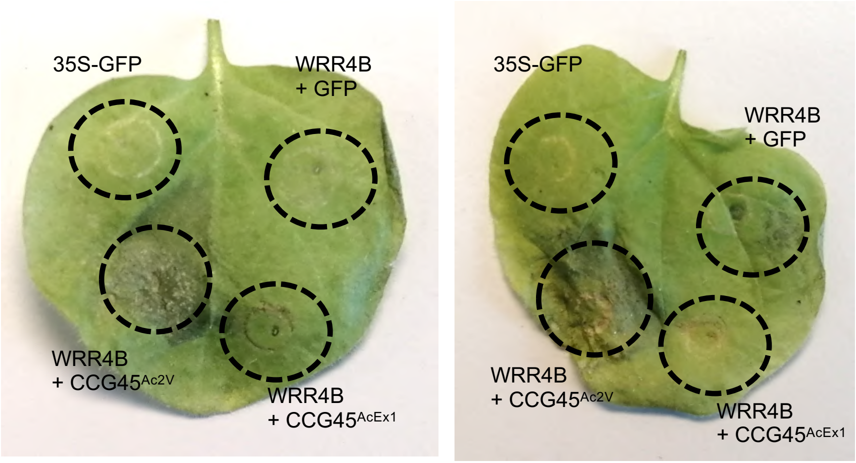

Fig. S8 CCG45^{Ex1} does not show enhanced *WRR4B*-dependent HR compared to CCG45^{Ac2V}
(a) Sequence alignment of AcEx1 and Ac2V variants of CCG45 shows highly divergent protein sequences. **(b)** Enhanced cell death observed in *N. benthamiana* leaves following co-expression of *WRR4B* with *CCG45*^{Ac2V} but not with *CCG45*^{AcEx1}. 35S-GFP was used as a negative control. *WRR4B* co-infiltrated with GFP is also included to show the weak autoimmune response. Two *N. benthamiana* leaf panels were photographed 4 dpi.

Fig. S9

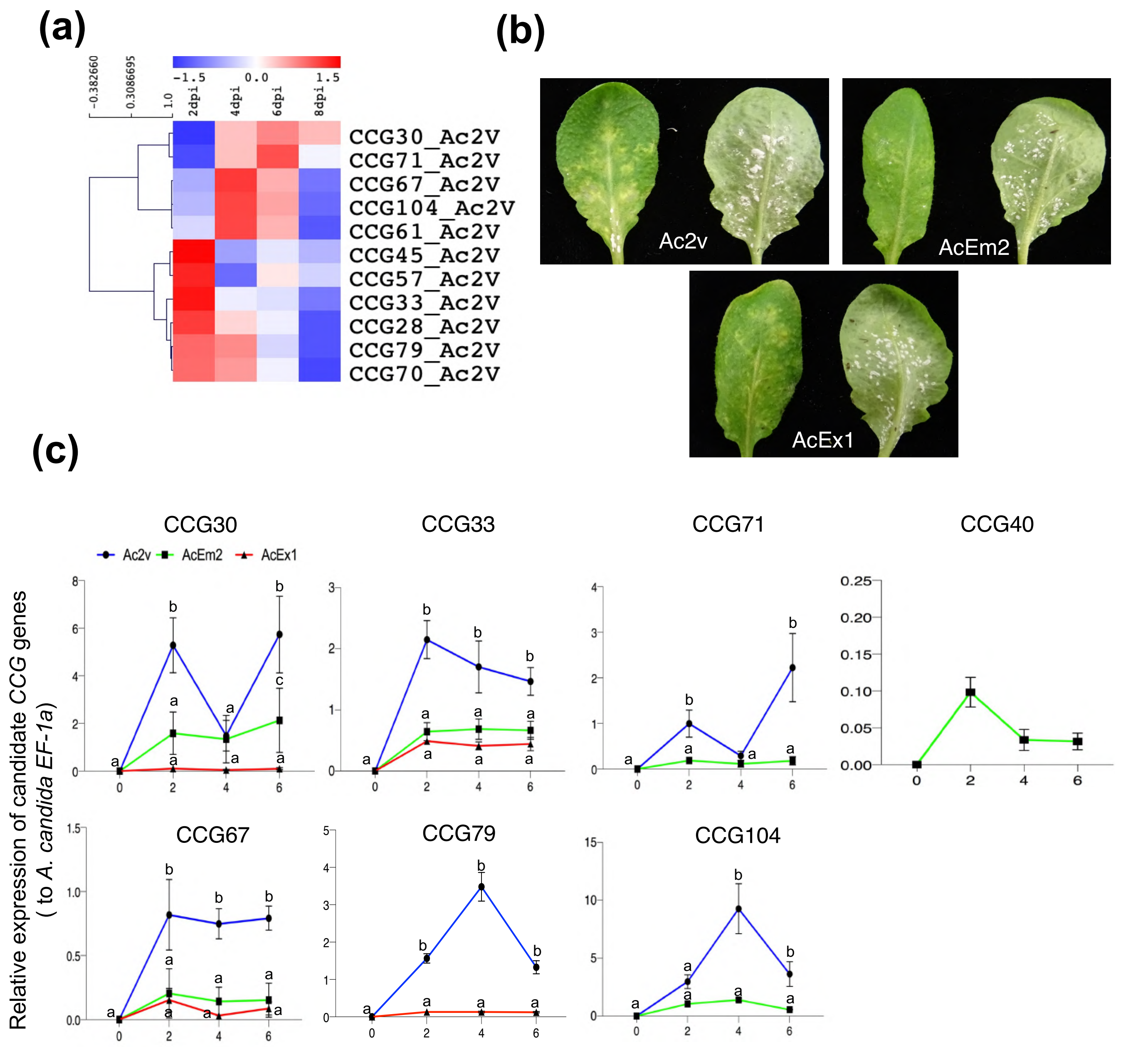

Fig. S9 Expression profiling of recognized *CCGs* by RNASeq and RT-qPCR analysis

(a) Expression profiles of *WRR4A*- and *WRR4B*-recognized *CCG*s from RNA-Seq data obtained over the consecutive time-points during infection stages of *A. candida* race Ac2V (Furzer *et al.,* 2021). The recognized CCGs show clear *in planta* expression suggesting their role during plant colonization.

(b) Disease phenotypes on Ws-*eds1 A. thaliana* plants upon infection *A. candida* races Ac2V, AcEm2 and AcEx1.

(c) Transcript levels of alleles of *WRR4A*-recognized *CCG*s at different timepoints following infection of Ws-*eds1* with different *A. candida* races. Expression was normalized to the Ac*EF1a* gene. Different letters indicate statistically significant differences between the different alleles tested (2-way ANOVA, Bonferroni´s multiple comparison test, $p < 0.05$). Error bars represent SD.

Fig. S10

(a)

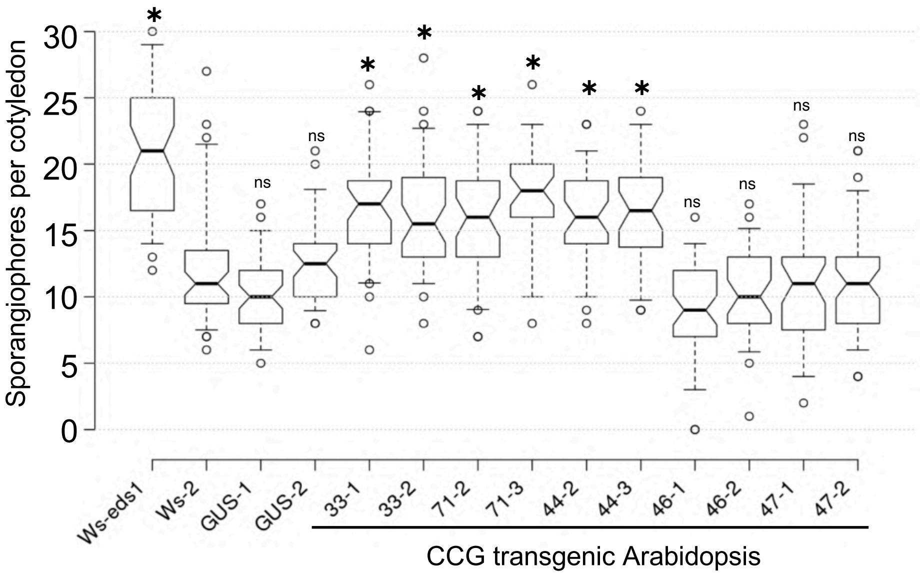

(b)

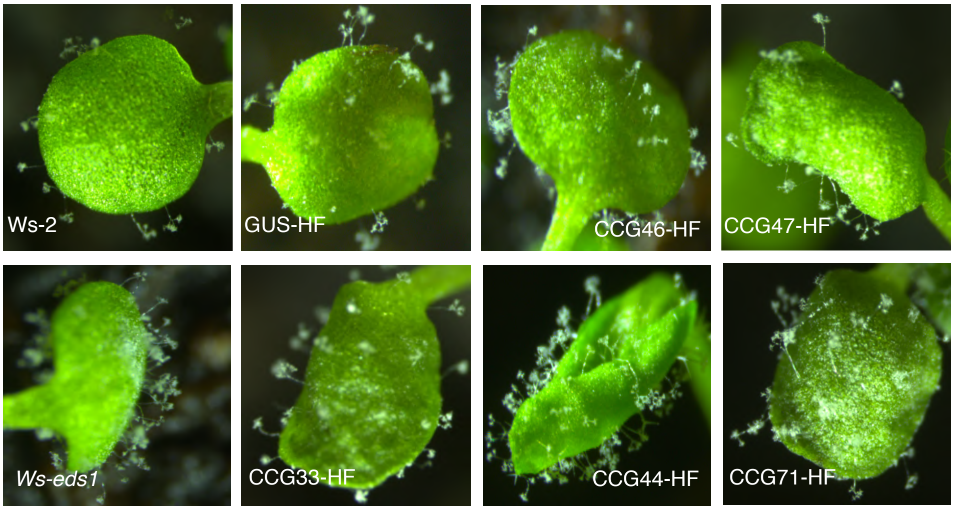

Fig. S10 Some CCG effectors elevate susceptibility to *H. arabidopsidis (Hpa)* Waco9 when constitutively expressed *in planta.*

(a) Two week-old soil-grown plants of Ws-2, *Ws-eds1* and transgenics expressing CCGs or GUS. were inoculated with *Hpa* Waco9 at a concentration of $1*10^5$ spores per ml and the number of sporangiophores were quantified 7 days after inoculation. Center lines show the medians. Whiskers extend to 5th and 95th percentile. Notches represent ± 1.58 interquartile range (IQR). Circles represent data points beyond 1.58 IQR. Asterisks indicate statistically significant differences as compared to the non-transformed control Ws-2 (one-way ANOVA; Bonferroni test, *,$P < 0.05$ or ns, nonsignificant, $P > 0.05$). n = 51, 51, 51, 40, 42, 44, 42, 33, 42, 36, 51, 38, 35, and 51 for each line.

(b) Enhanced susceptibility to *Hpa* Waco9 in seven-day-old *CCG*-expressing, GUS- control or non transformed background and positive control plants. Plants inoculated with *Hpa* Waco9 were photographed at 7 dpi. Representative images are shown.

Supplemental References

Furzer OJ, Cevik V, Fairhead S, Bailey K, Redkar A, Schudoma C, MacLean D, Holub EB, Jones JDG. 2022. An improved assembly of the *Albugo candida* Ac2V genome reveals the expansion of the "CCG" class of effectors. *Molecular Plant-Microbe Interactions* **35**(1): 39-48.

Segretin ME, Pais M, Franceschetti M, Chaparro-Garcia A, Bos JI, Banfield MJ, Kamoun S. 2014. Single amino acid mutations in the potato immune receptor R3a expand response to Phytophthora effectors. *Mol Plant Microbe Interact* **27**(7): 624-637.
