# Supplementary material for: The Arabidopsis WRR4A and WRR4B paralogous NLR proteins both confer recognition of multiple Albugo candida effectors
Source: New Phytol. 2022 Aug 7;237(2):532–47. doi: 10.1111/nph.18378 (PMC10087428; doi:10.1111/nph.18378)
Supplement: Supplementary file 5 — Table S3 Allelic status and presence/absence variation of the WRR4A‐recognised CCGs across different Albugo candida isolates. Table S4 Allelic status and presence/absence variation of the WRR4B‐recognised CCGs across different Albugo candida isolates. Please note: Wiley Blackwell are not responsible for the content or functionality of any Supporting Information supplied by the authors. Any queries (other than missing material) should be directed to the New Phytologist Central Office. [file NPH-237-532-s003.pdf]

New Phytologist Supporting Information

Article title: **The Arabidopsis *WRR4A* and *WRR4B* paralogous NLR proteins both confer recognition of multiple *Albugo candida* effectors**

Authors: Amey Redkar, Volkan Cevik, Kate Bailey, He Zhao, Dae Sung Kim, Zhou Zou, Oliver J. Furzer, Sebastian Fairhead, M. Hossein Borhan, Eric B. Holub and Jonathan D.G. Jones

Article acceptance date: 5 July 2022

+ present as full length, **-** completely absent **Ψ** truncated

Effector	Length (aa)	AcNc2	Ac2v	AcEx1	AcEm2	Ac7v	AcBoT
CCG28	563	+	+	Ψ (226)*	+	+	+
CCG30	550	+	+	+*	+	+	+
CCG33	678	+	Ψ (242)	+*	+	+	+
CCG71	589	+	+	-	+	+	+
CCG40	216	+	Ψ (54)	Ψ (2)	+	+	Ψ (169)
CCG67	592	+	+	+*	+	+	Ψ (489)
CCG79	796	-	+	+*	-	+	+
CCG104	658	+	+	-	+	+	+

Table S3: Allelic status and presence/absence variation of the WRR4A-recognized CCGs across different *A. candida* isolates

Overview of the variation of all the WRR4A-recognized CCG variants, within different *A. candida* isolates. The CCG variants across different *A. candida* isolates are denoted by (+) when present in full length, (-) when it is absent in a particular isolate and (**Ψ**) when truncated with an early stop codon. The (*) indicate low expression of the candidate CCG in that particular isolate of *A. candida.*

+ present as full length, **-** completely absent **Ψ** truncated

Effector	Length (aa)	AcNc2	Ac2v	AcEx1	AcEm2	Ac7v	AcBoT
CCG45	721	Ψ# (0)	+	+	Ψ# (0)	+	Ψ (312)
CCG57	705	Ψ (157)	+	Ψ (157)	Ψ (157)	+	+
CCG61	600	Ψ (115)	+	Ψ (115)	Ψ (115)	+	+
CCG70	597	-	+	-	-	+	+

Table S4: Allelic status and presence/absence variation of the WRR4B-recognized CCGs across different *A. candida* isolates

Overview of the variation of all the WRR4B-recognized CCG variants, within different *A. candida* isolates. The CCG variants across different *A. candida* isolates are denoted by (+) when present in full length, (-) when it is absent in a particular isolate and **(Ψ)** when truncated with an early stop codon. # Mutation occurred in translation start site.
